# The Transcriptome of Pig Spermatozoa, and Its Role in Fertility

**DOI:** 10.3390/ijms21051572

**Published:** 2020-02-25

**Authors:** Manuel Alvarez-Rodriguez, Cristina Martinez, Dominic Wright, Isabel Barranco, Jordi Roca, Heriberto Rodriguez-Martinez

**Affiliations:** 1 Department of Biomedical & Clinical Sciences (BKV), BKH/Obstetrics & Gynaecology, Faculty of Medicine and Health Sciences, Linköping University, SE-58185 Linköping, Sweden; cristina.martinez-serrano@liu.se (C.M.); heriberto.rodriguez-martinez@liu.se (H.R.-M.); 2 Department of Physics, Chemistry and Biology, Faculty of Science and Engineering, Linköping University, SE-58183 Linköping, Sweden; 3 Biotechnology of Animal and Human Reproduction (TechnoSperm), Department of Biology, Institute of Food and Agricultural Technology, University of Girona, 17003 Girona, Spain; isabel.barranco@um.es; 4 Department of Medicine and Animal Surgery, Faculty of Veterinary Medicine, International Campus for Higher Education and Research “Campus Mare Nostrum”, University of Murcia, 30100 Murcia, Spain; roca@um.es

**Keywords:** transcriptomics, microarrays, bioinformatics, spermatozoa, fertility, pig

## Abstract

In the study presented here we identified transcriptomic markers for fertility in the cargo of pig ejaculated spermatozoa using porcine-specific micro-arrays (GeneChip^®^ miRNA 4.0 and GeneChip^®^ Porcine Gene 1.0 ST). We report (i) the relative abundance of the ssc-miR-1285, miR-16, miR-4332, miR-92a, miR-671-5p, miR-4334-5p, miR-425-5p, miR-191, miR-92b-5p and miR-15b miRNAs, and (ii) the presence of 347 up-regulated and 174 down-regulated RNA transcripts in high-fertility breeding boars, based on differences of farrowing rate (FS) and litter size (LS), relative to low-fertility boars in the (Artificial Insemination) AI program. An overrepresentation analysis of the protein class (PANTHER) identified significant fold-increases for C-C chemokine binding (GO:0019957): CCR7, which activates B- and T-lymphocytes, 8-fold increase), XCR1 and CXCR4 (with ubiquitin as a natural ligand, 1.24-fold increase), cytokine receptor activity (GO:0005126): IL23R receptor of the IL23 protein, associated to JAK2 and STAT3, 3.4-fold increase), the TGF-receptor (PC00035) genes ACVR1C and ACVR2B (12-fold increase). Moreover, two micro-RNAs (miR-221 and mir-621) were down- and up-regulated, respectively, in high-fertility males. In conclusion, boars with different fertility performance possess a wide variety of differentially expressed RNA present in spermatozoa that would be attractive targets as non-invasive molecular markers for predicting fertility.

## 1. Introduction

Just minutes after natural mating or artificial insemination (AI) boar spermatozoa reach the sow oviduct [1], and increasing evidence indicates an interaction occurs between the semen and the lining epithelium of the female genital tract that initiates changes aiding the establishment and maintenance of pregnancy [2]. Transcriptomic changes in the pig uterus and oviduct in the presence of semen have been previously described [3,4]. Semen starts an inflammatory response by the female [5], and further induces changes in the expression of immune-related genes [4], apparently leading to the establishment of an immune tolerance status to paternal antigens by the female immune system [6]. This cross-talk between semen and the female genital tract seems initiated by signals originating from components of the seminal plasma (proteins, cytokines, extracellular vesicles) and spermatozoa (proteins, associated exosomes, RNAs) [4]. 

In mammals, spermiogenesis implies crucial changes of cell differentiation in round spermatids that leads, among other specific modifications, to the loss of most of their cytoplasm before becoming a testicular spermatozoon that soon moves from the seminiferous epithelium towards the epididymis. Further maturational changes occur in the epididymis, including compaction of the nuclear genome [7], that leads to the general belief that the ejaculated spermatozoon is unlikely to perform transcription and translation until its nucleus decondenses following fertilization [8]. However, the presence of RNA, maybe only representing remnants of spermatogenesis, has been described in boar spermatozoa [9], including non-coding RNA and miRNAs [10] (short non-coding RNA molecules that regulate gene expression via post-transcriptional gene silencing, chromatin-dependent gene silencing or RNA activation [11]). The presence of different miRNAs has been reported in both epidydimal and ejaculated mammalian spermatozoa [12,13]. In boars, miRNA profiling has been previously reported in testis, epididymis and ejaculated spermatozoa [14,15]. An increased expression of certain miRNAs in boar spermatozoa (let-7a, -7d, -7e, and miR-22) has been correlated with low sperm motility [16]. However, we still lack conclusive evidence of the origin [9], and the precise roles of these miRNAs in spermatozoa, including their impact on fertilization and the subsequent development of the zygote, ultimately affecting fertility.

In this relation, decreased levels of miR-10b, and miR-135b in asthenospermic human sperm samples compared to healthy controls [17] and other miRNAs has been associated with asthenozoospermic, teratozoospermic, and oligozoospermic populations [17,18]. However, to the best of our knowledge, similar studies of differential expression of miRNAs in relation to fertility performance are not available in other species including pigs, a species increasingly used as an animal-model for human fertility due to its similarities [19]. The porcine model allows us to not only analyse the farrowing rate and litter size but to correlate it with a wide range of sperm quality parameters or even differential RNA sperm load. Moreover, in pigs, AI has, in most countries, replaced natural mating and breeding boars are continuously renewed after a certain number of AIs. Thus finding suitable fertility markers in semen is an ongoing strategic goal, since it would reduce the numbers of less fertile boars being used and allow for enhanced selection of the most fertile candidates [20].

The same reasoning would also apply to markers based on gene expression, even when spermatozoa are supposed to be transcriptionally silent (with the exception of their mitochondria [21,22]). In fact, the total RNA cargo of boar spermatozoa present in the ejaculate and selected for robustness has been analyzed via RNA-seq [23], and indicates the presence of readily available transcripts.

The present study aimed therefore to determine the relative load (in terms of expression levels) of miRNAs and mRNAs in ejaculated boar spermatozoa, including the relation of any differential RNA cargo with fertility characteristics post AI. Microarray-based technology was used to study either (i) relative abundance of miRNAs on boar spermatozoa and (ii) total RNA differential expression between high fertile and low fertile boars. The hypothesis tested was that the available RNA-cargo in ejaculated spermatozoa includes specific transcripts whose level of expression is enriched in high-fertility breeding boars and thus could be valuable biomarkers for fertility.

## 2. Results

### 2.1. Experiment 1: Screening of Sperm miRNAs in the Sperm-rich Fraction (SRF) of the Boar Ejaculate

Total RNA was extracted from spermatozoa retrieved from the SRF of the ejaculate of healthy mature boars (*n* = 3), and then analyzed with the GeneChip™ miRNA 4.0 Array. Of the 326 pig (*Sus scrofa*)-specific miRNAs included in the array, 10 miRNAs had an expression of more than 10 average expression units (log 2 average expression = >1031 times expression from the basal-normalized signal), with these being miR-1285, miR-16, miR-4332, miR-92a, miR-671-5p, miR-4334-5p, miR-425-5p, miR-191, miR-92b-5p and miR-15b (Table 1). A total of 71 miRNAs were expressed between 5 and 10 average expression units and 245 miRNAs showed only <5 average expression units (Supplementary Table S1).

### 2.2. Experiment 2. Differential Cargo of RNAs in Ejaculated Spermatozoa from Breeding Boars with High- or Low-fertility (as Farrowing Rate and Litter Size) after Artificial Insemination (AI)

**Figure 1:**
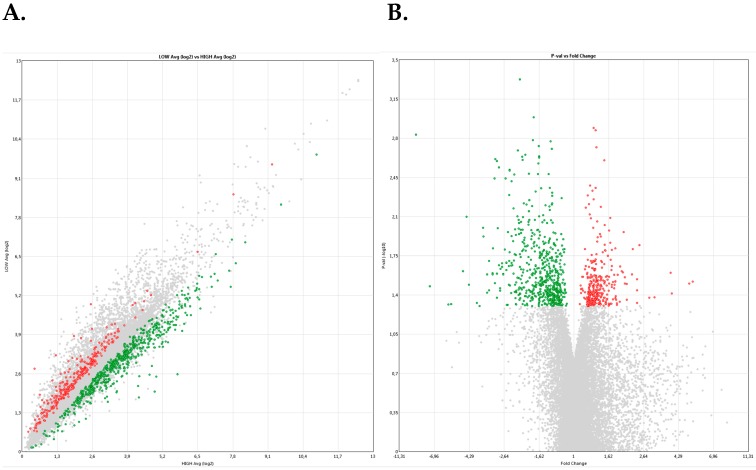
(**A**) Scatter plot and (**B**) Vulcano plot of differential gene expression using a microarrays platform (GeneChip^®^ Porcine Gene 1.0 ST Array) of 521 transcripts (347 transcripts up-regulated and 174 transcripts down-regulated) in high-fertile relative with low-fertile males. Annotation of the full gene name for the transcript and up- (red color-positive fold change) and down-regulation (green color-negative fold change in high-fertile males in comparison to low-fertile males. The Porcine Genome Array provides comprehensive coverage of the S. scrofa transcriptome. The array contains 23,937 probe sets that interrogate approximately 23.256 transcripts from 20.201 *Sus scrofa* genes. The sequence information for this array was selected from public data sources including UniGene GenBank^®^ mRNAs, and GenBank porcine mitochondrial and rRNA sequences.

The Bioinformatic analysis of the normalized data collected from the gene expression analysis using the GeneChip^®^ Porcine Gene 1.0 ST Array, using a Fold-change threshold value of ± 1 (p-value < 0.05) identified up to 521 differentially expressed genes (DEGs), among which 374 were up-regulated in high-fertility (*n* = 4) as compared to low-fertility (*n* = 3) males (Figure 1 and Supplementary Table S2). 

When we performed a more stringent analysis of these DEGs (Table 2), 67 out of 347 up-regulated genes in high-fertility boars had a fold-change higher than 2, whereas 10 out of 174 down-regulated genes in high-fertility boars had a fold-change lower than −2. 

**Figure 2:**
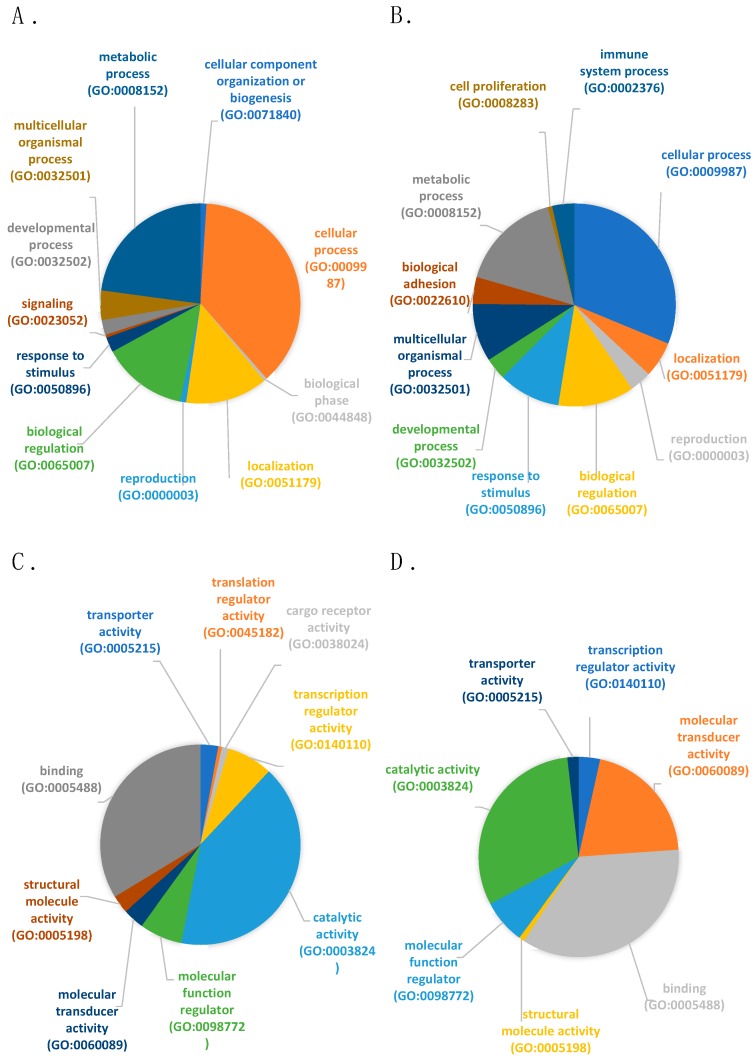
Differentially expressed genes (DEGs) classification (PANTHER) according to 1) biological process (**A**). up-regulated genes in high-fertile boars and (**B**). down-regulated genes in high-fertile boars; and 2) molecular function (**C**). up-regulated genes in high-fertile boars and (**D**). down-regulated genes in high-fertile boars.

Figure 2 depicts a PANTHER analysis of the biological functions of the differentially expressed genes (DEGs) (Figure 2A,B). The majority of the functions attributed for both up- and down-regulated genes were: cellular processes (GO:0009987), metabolic processes (GO:0008152) and biological regulation (GO:0065007). Interestingly, genes related to biological adhesion (GO:0022610; CDH10, CDSN, ITGB8, ANGPTL1, CTNNA3) and cell proliferation (GO:0008283; CCNJ) were only found in down-regulated genes in high-fertility boars whereas related genes to signaling (GO:0023052; FAM109A) and cellular component organization or biogenesis (GO:0071840; KRI1, ZNHIT6) were only identified in up-regulated genes in high-fertility boars.

In terms of molecular function (Figure 2C,D), both dysregulated groups of genes presented and overrepresentation of genes mainly involved in binding (GO:0005488) and catalytic activity (GO:0005488). Genes related to cargo receptor activity (GO:0038024; CXCL16) and translation regulator activity (GO:0045182; CPEB3) were, on the other hand, only found in up-regulated DEGs in high-fertility boars. 

**Figure 3:**
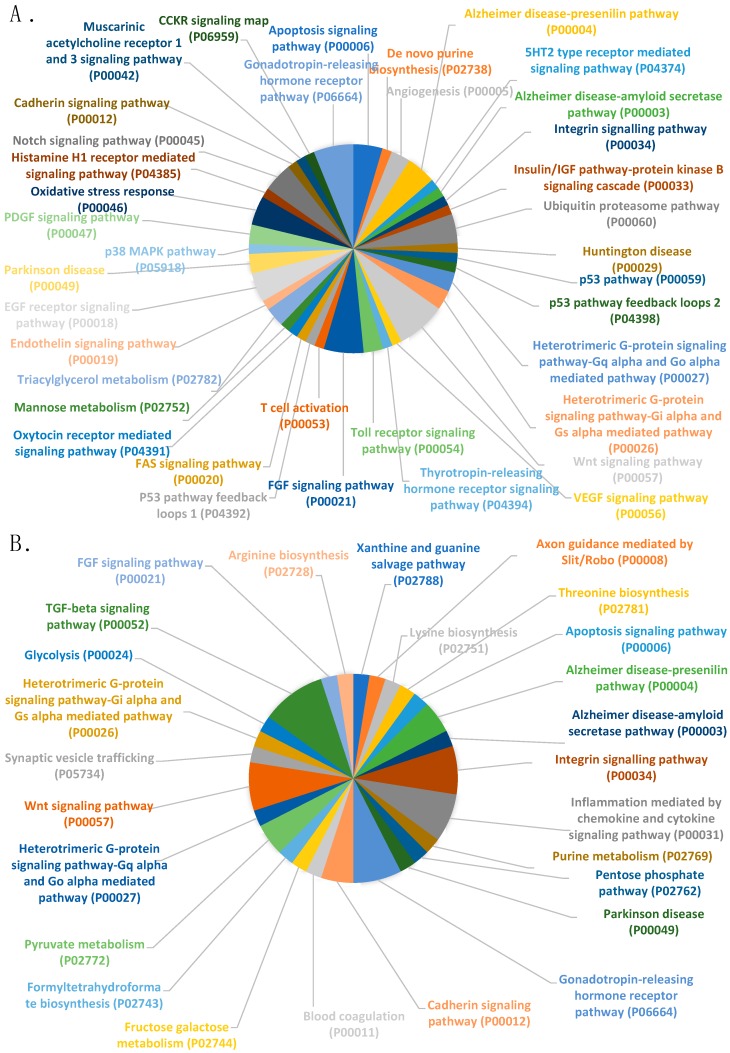
Differential expressed genes (DEGs) classification (PANTHER) according to pathway (**A**). up-regulated genes in high-fertile boars and (**B**). down-regulated genes in high-fertile boars.

Figure 3 summarizes the analysis of the pathways in both groups of DEGs (up- (Figure 3A) and down-regulated (Figure 3B) genes) in high-fertility boars, detailed pie-chart include a large variety of functions common to up- and down-regulated genes in high-fertile boars. 

An over-representation analysis (PANTHER) (Table 3) allowed us to restrict the number of pathways according to their representation significance (*p* < 0.05) relative to the expected genes present in the genome of *Sus scrofa*.

Finally, the analysis of a plausible interconnection between target genes of those miRNAs detected in experiment 1 (SRF-derived spermatozoa, with more than 10 average expression units) and the DEGs in the PORGENE-array in experiment 2 is presented in Table 4. Only a few genes, mostly up-regulated in high-fertility males, appear to be potentially shared. In this regard, results from DEGs in terms of miRNA dysregulation found only miR-615 up- and miR-221 to be down-regulated in high-fertility boars relative to the low-fertility boars (Supplementary Table S2).

## 3. Discussion

The microarray platform analysis used here and, in particular the multispecies miRNA array, have allowed us to define the relative abundance or cargo for large numbers of non-coding small RNAs present in spermatozoa retrieved from the SRF of boar ejaculates. An alternative would have been to use qPCR, but the latter is highly reliant on the number and selection of housekeeping genes [24]. The microarray platform is more robust and stable than a single qPCR because it is normalized against the total expression of the entire transcriptome that is measured in the array. The information provided by the microarray has been widely studied in terms both of the spermatogenesis process, in which the cytoplasm is reduced to a minimal portion, and the function of this restricted load plays on further endometrium receptivity and even on embryo development.

Only a few miRNAs out of the detected 326 pig-specific miRNAs were abundant. Of those in abundance, the miR-1285 has been related to sperm production, and considered inhibited by 17β-estradiol, the latter (estradiol) also promoting AMPK phosphorylation [25]. Moreover, this miR-1285 has been previously implicated in oxidative stress, with a response seen in human retinal pigment epithelial using a hydrogen peroxide challenge as an *in vitro* model for the induction of oxidative stress [26]. Another highly abundant miRNA, miR-15/miR-16 seems to suppress the TGF-β Signaling Pathway (a key superfamily cytokines that promotes apoptosis), mainly through the inhibition of the expression of endogenous Smad3 and ACVR2A proteins [27]. This is likely related to our previous finding which demonstrated the differential expression of immune regulatory genes in the porcine female genital tract in the presence of spermatozoa and seminal plasma [4]. The miRNA miR-4332 is a key regulator of lipid deposition found in pig muscle tissue [28] but has not previously been identified as a candidate for any known function directly related to reproduction. Increased levels of expression of miR-92 relate to decreases in the estrogen receptor β1 [29] and also suppresses proliferation and induces apoptosis by targeting EP4/Notch1 Axis, regulated by the NF-kB pathway [30]. Both of these miRNAs might be coupled to sperm survival and the triggering of certain responses in the female through miR-92 release from the spermatozoon. The miRNA miR-671-5p is an important inhibitor of cell proliferation and inductor of apoptosis by targeting *URGCP* (upregulator of cell proliferation) [31] and also the suppression of *FGFR2* [32] (gene up-regulated in high-fertility boars in our results). Although this gene *FGFR2* has been defined as non-essential for spermatogenesis and fertility in mice [33], it could play a different and more essential role in boar spermatozoa, with the potential of being used as a fertility biomarker. MiR-4334 is found in milk exosomes reduced lipopolysaccharide (LPS)-induced apoptosis via the p53 pathway; LPS is a bacterial endotoxin that induces inflammation [34]. Its presence could indicate a further regulation of this process in the female genital tract, induced via a sperm mediator. Mir-425-5p negatively regulates *E2F6*, a key regulator of the cell cycle and apoptosis [35] and, together with miR-4332, is linked to a negative regulation of the differentiation and proliferation of intramuscular pre-adipocytes [36]. Thus, the relative abundance of these two regulators should be studied to link the anti-apoptotic signaling from the spermatozoa to the female genital tract. Moreover, miR-425 and *IL-23* have been correlated [37] and its receptor (IL-23R) was down-regulated in our comparison of high- vs. low-fertility boars. *IL-23* play critical roles in the induction of Th17 differentiation and governs local inflammation [38], so it might be possible that a reduction in its receptor expression could translate in a reduction of the local immune reaction in the female to the presence of semen. Finally, among these overexpressed miRNAs, miR-191, in combination with miR-425, increases cell proliferation, survival and migration [39], potentially related to the travel of the spermatozoon through the entire female genital tract until the site of fertilization in the mid-ampulla of the oviduct or via repressive signals to the female genital tract during this sperm progression. It has been also described as key activator of the NF-κB signaling pathway [40] as well as a regulator of the estrogen receptor [41] and an inhibitor of the Wnt pathway [42]. Interestingly, some studies in humans have reported the reduced levels of miR-191 in the semen of infertile human patients [17,18]. 

The main focus of the second experiment was to identify, via microarray analysis, which transcripts were differentially expressed in ejaculated spermatozoa from breeding boars, highly selected for fertility after artificial insemination in commercial programs. The boars selected had fertility data based on more than 100 artificial inseminations (up to 540) per boar. The analyses of fertility resulted in two clear subgroups according to their deviation in farrowing rate (FR) and litter size (LS) from the average direct boar effect recorded by the boar population of the same genetic line: high-fertility boars (FR > 0.45 and a LS > 0.15); and low-fertility boars (FR < −0.12 and LS < −0.18). These fertility results show the true differences in the fertile potential of boars, since they were specifically recorded to show the direct effect of the boar on fertility, which was possible by using the statistical model developed by Broekhuijse et al. [43], and represent a very strong selection of individuals who would scape conventional screening of semen quality, which is continually done in breeding AI-programs. The microarrays used allow for the identification of more than 25,000 transcripts from total RNA, including isoforms, of *Sus scrofa* annotated genes and also of non-coding RNAs. The overall results showed differential gene expression with 347 up-regulated genes and 174 down-regulated genes in high-fertility boars relative to low-fertility boars. These findings are relevant based on the fact that the boars with different specific fertility included in the present study were breeding boars, highly selected for sperm quality, a pre-requisite for inclusion in programs of artificial insemination. All boars had normal semen quality, in terms of sperm numbers, sperm motility, sperm morphology and absence of inflammatory; contrasting with other species as human, or even other animal classes [44] that had been explored for the specific presence of miRNAs in individuals with clear differences in sperm quality and the influence of high- or low-sperm quality on progeny, often caused by specific spermatogenic impairment [45]. Of interest, these boars, deviating in true fertility but having normal semen quality, had differences in gene expression that ought to be relevant as future biomarkers. Two miRNAs: miR-615 appeared up-regulated while the miR-221 was down-regulated in high-fertility boars. The miR-615 is considered an inhibitor of apoptosis targeting EGFR [31], and it has been studied as being active in the presence of epidermal growth factor receptor (EGFR) in spermatozoa, as well as having a role in the transactivation by direct activation of the G-protein coupled receptor (GPCR), as well as being involved in sperm capacitation and in the acrosome reaction [46]. Thus, it could be a possible link between capacitation and the miR-615 expression. On the other hand, The miR-221 has been related to *Wnt2*, *BDNF* and *CREB*-related genes [47] and ties in to a previous result by our group describing the downregulation of *CREB3L2* (p<0.05) in the uterus and utero-tubal junction in the porcine female genital tract in response to both mating and artificial insemination. Finally, it is also related to PI3K-Akt and the estrogen signaling pathway [4]. 

Overall (See Table 2 and Supplementary Table S2, DEGs), we have found several transcripts related to membrane channels that were up-regulated in high-fertility boars: *KCNA3* (potassium channel, voltage gated shaker related subfamily A, member 3), *KCNIP3* (Kv channel interacting protein 3, calsenilin), *KCNH4*; *LOC100620256* (potassium channel, voltage gated related subfamily H, member 4; potassium voltage-gated channel subfamily H member 4-like), LOC102159765 (voltage-dependent P/Q-type calcium channel subunit alpha-1A-like), KCTD9 (potassium channel tetramerization domain containing 9), *CNGA4* (cyclic nucleotide gated channel alpha 4). Among these channels, only *KCNA3* has been characterized in rat testis as well as identified not only in the cytoplasm of primary spermatocytes and post-meiotic elongating spermatids, but also in the head and tails of epididymal spermatozoa [48]. Whether these channels are directly related to capacitation is still unknown, but the role of this gene should be examined in future studies in this context, in particular at the protein level. Finally, *CATSPERG* (catsper channel auxiliary subunit gamma) is up-regulated whereas the *CATSPERB* (catsper channel auxiliary subunit beta) was down-regulated in high-fertility boars. These two last transcripts are subunits of catsper, a channel involved in boar sperm motility during *in vitro* capacitation [49]. The CATSPER protein complex contains *CATSPERB* and *CATSPERG,* the latter associated with *CATSPER1* [50] and the inhibition of *CATSPER* is linked to an increase on a reactive oxygen species (ROS) generation [51] and it is involved in key processes of mammalian fertilization by Calcium signaling [52].

Moreover, a representative number of transcripts included in the list of our DEGs were related to Zinc finger proteins (ZNF), one of the most abundant group of proteins with a wide variety of functions, including transcriptional regulation, ubiquitin-mediated protein degradation, signal transduction, actin targeting, DNA repair and cell migration [53]. The majority of the ZNF-related transcripts were up-regulated in high-fertility boars: *LOC100739821* (zinc finger MIZ domain-containing protein 2), *ZNRF4* (zinc and ring finger 4), *LOC100512674* (zinc finger protein 367), *LOC100511053*; *LOC100624021* (zinc finger protein PLAGL2), *ZFAND3* (zinc finger, AN1-type domain 3), *ZNF25* (zinc finger protein 25), *ZDHHC7* (zinc finger, DHHC-type containing 7), *ZNF268* (zinc finger protein 268), *ZNHIT6* (zinc finger, HIT-type containing 6), *ZNF568* (zinc finger protein 568), LOC102163983 (zinc finger protein 341). Among these, *ZDHHC7* is directly involved in signaling via membrane-localized estrogen receptors (ER) [54] and the ER interacts directly with the Phosphatidylinositol-3-OH Kinase/Akt Pathway [55]. We conjecture that this up-regulation in the boar spermatozoa from high-fertility males could interact with the ER in the female genital tract. It is also relevant for the role of *ZNF568* in a later event, being expressed in 3-5 week old embryos and potentially is related to embryogenesis [56]. In contrast, only a few of these genes were found to be down-regulated in high-fertility boars: *ZNF283* (zinc finger protein 283), *FEZF2*; *LOC100622205* (FEZ family zinc finger 2; fez family zinc finger protein 2), *GLI1*; LOC100621250 (GLI family zinc finger 1; zinc finger protein GLI1-like). Interestingly, down-regulation in high fertility males is in accordance with a previous study on GLI1 up-regulation in transgenic mice leading to a blockage of spermatogenesis in pachytene [57]. This gene is directly involved in the sonic hedgehog (Shh) signaling pathway, with important roles in pattern orientation in the developing embryo [58].

Transcription factors are also represented among our results of DEGs. Not only are they up-regulated in high-fertility boars: *LOC100152461*; *LOC100627430*; *NFYA* (nuclear transcription factor Y subunit alpha; nuclear transcription factor Y, alpha), *TCF21* (transcription factor 21), *LOC100524199*; *LOC100620736* (transcription factor 25), *MBTPS2* (membrane-bound transcription factor peptidase, site 2), *MTF2* (metal response element binding transcription factor 2), but some are also down-regulated in high-fertility boars: *UBTFL1* (upstream binding transcription factor, RNA polymerase I-like 1).

The matrix metalloproteinases (ADAM) are involved in several processes such as cell adhesion and immune related signaling, including the activation of TNF-alfa and generation of the active forms of Epidermal Growth Factor Receptor (*EGFR*) [59]. In fact, the relevance of this *ADAM* gene has been previously described by our group, mainly in terms of biological adhesion but also in terms of pH regulation of the utero tubal junction in sows [60,61]. In the present study, we identify down-regulation in high-fertility boars of *ADAM7* (ADAM metallopeptidase domain 7) and ADAM29 (ADAM metallopeptidase domain 29). *ADAM7* in mouse spermatozoa is required for normal fertility [62], and here we its presence and the decreased abundancy in high-fertility spermaozoa. Both ADAMs have been linked to the increase of adhesion capacities of extracellular matrix proteins [63]. Whether this increase in adhesion is related to the progression of spermatozoa through the female genital tract is yet to be determined. Other relevant immune-related transcripts that appeared down-regulated in high-fertility boars were *LOC102162067* (interleukin-6 receptor subunit alpha-like), *IL23R* (interleukin 23 receptor), *IFN-DELTA-4*; *LOC100736862* (interferon-delta-4; interferon tau-11-like), IFNK (interferon, kappa), *LOC100736882* (interferon lambda receptor 1-like) and *IFIT3* (interferon-induced protein with tetratricopeptide repeats 3), however similar genes were also found to be up-regulated in high-fertility boars: *IRF3* (interferon regulatory factor 3) and *ISG20L2* (interferon stimulated exonuclease gene 20kDa-like 2).

In order to categorize the overall DEGs resulted from our study, we performed a biological function analysis (PHANTER) of the DEGs which resulted in a differential function depending on the fertility performance of the boars. Thus, biological adhesion-related genes (GO:0022610; *CDH10, CDSN, ITGB8, ANGPTL1, CTNNA3*) were found only to be down-regulated in high-fertility boars. In contrast, genes related to signaling (GO:0023052; *FAM109A*) and cellular component organization or biogenesis (GO:0071840; *KRI1*, *ZNHIT6*) were only identified as up-regulated in high-fertility boars. Cadherin-10 (*CDH10*), the type II classical cadherin, a protein binding molecule in terms of molecular function and is used as a biomarker in the prognosis of certain types of cancer [64], whilst *CDSN* is involved in skin immune-related disease [65]. Whether the down-regulation of both these genes in our study might be related to a decrease in the attachment of the spermatozoa to certain sections of the female tract remains to be studied. *ITGB8* is involved in the endometrial receptivity through VAV-RAC1 signaling axis via FAK to facilitate the endometrial epithelial cell receptivity for the attachment of blastocyst [66] and, surprisingly, is less abundant in high-fertility boars. *ANGPTL1,* Angiopoietin-like proteins is involved in several processes from lipid and glucose metabolism to inflammation [67]. The last of this biological adhesion group is *CTNNA3* (alpha-T-catenin), whose gene dosage compensation of *CTNNA3* and *p57KIP2* in the placenta shares a conserved regulatory mechanism that correlates with an early step in trophoblast determination [68]. Regarding cell proliferation, the cyclin J (*CCNJ*), down-regulated in high-fertile boars, is involved in cell cycle regulation, but without any apparent described function in spermatogenesis or fertility performance. Regarding up-regulated genes in high-fertility boars, *ZRI1* permits DNA damage-induced apoptosis of cells in the germline by an unknown cell non-autonomous mechanism [69] and *ZNHIT6* is required for the maintenance of correct protein folding under heat stress conditions [70].

If we categorize according to molecular function, one of the main findings was the cytoplasmic polyadenylation element-binding protein 3 (*CPEB3*), a translational regulator involved in long-term memory storage maintenance in synaptic connections in neurons [71], which has also been involved in EGFR axis regulation [72] and also in SUMOylation [73]. SUMOylation is a process that controls the activity of pre-existing proteins through a reversible post-transcriptional modification [74]. In addition, decidualization renders the endometrium transiently receptive to an implanting blastocyst although the underlying mechanisms remain incompletely understood, but *CXCL16* appears to be an initiator for the molecular crosstalk that enhances decidualization of the endometrial stromal cells [75], despite this process as such has not been described in pig. Nevertheless, this fact is certainly in agreement with our results, observing up-regulation of this gene in high-fertility boars, but whether a “remodeling” or decidualization-like process is present in pig remains to be studied.

A further analysis of the overrepresentation analysis in terms of molecular function reveals an important component on chemokine binding (GO:0019957). Regulatory T (Treg) cells facilitate maternal immune tolerance of the hemi-allogeneic conceptus in early pregnancy. In this regard, seminal fluid induced expression of mRNA encoding the Treg chemokine *CCL19* (*MIP3beta*), which acts through the *CCR7* receptor to regulate Treg cell recruitment and retention in peripheral tissues [76]. Our results showed a decrease in abundancy of the *CCR7* receptor in high-fertile boars. In addition, *CXCR4*, is the main G-coupled receptor of *CXCL12*, involved in several diseases in the female reproductive tract, mainly through inflammatory processes [77]. In agreement with this, the lower abundancy of this receptor on high-fertile boars, perhaps in relation to a reduced inflammatory response of the female genital tract in response to the entrance of spermatozoa with a lower load of this transcript.

We have also observed in our analysis of protein class overrepresentation, that the *ACVR1C* and *ACVR2B* were down-regulated in high-fertility males. Activin affects many aspects of cellular development, including those essential for reproductive fitness and, in fact, activin receptor transcript levels also change, with *Acvr1* (encoding *ALK2*) and *Acvr2b* (*ActRIIB*) significantly higher and lower, respectively in the proliferation of mouse testis [78].

Finally, in an attempt to address a prospective functional approach to the high-expressed miRNAs, we contrasted the target genes of the most abundant miRNA (with a target match of more than 0.9. miRbase and miRDB [79]), and found several genes of interest that were mostly up-regulated in the high-fertility boars. In Table 4, as detailed in the results section, we found target genes differentially expressed in relation with miR-16, miR-92a and miR-15b. Interestingly, up-regulation of certain genes (*UBQLNL, CPEB3, DESI1* and *ATG9A*) and down-regulation in high- relative with low-fertile boars were found both link to miR-16 and miR15b. In common with miR-92b we identified the transcript *CPEB3*, and the specific genes related solely to this miRNA: *TCF21*, *PDZD8*. Finally, in relation with miR-16, the gene *BAG4* appeared up-regulated in the DEGs analysis performed with microarrays. The *UBQLNL* is a testis-specific gene, not indispensable due to a compensatory mechanism in mice, but involved in the elongation of spermatids and found up-regulated in high-fertile boar samples [80]. It is a member of the ubiquilin gene family, highly conserved in eukaryotes and with high expression levels in testis, and mainly in the “spermatid” stage [81]. In relation with the already mentioned SUMOylation process, our results of DEGs included the up-regulation in high-fertility boars of a SUMO1 activating enzyme subunit 1 (*SAE1*), essential for the maintenance of hematopoietic stem/progenitor cell in zebrafish [82] and also *DESI1* (desumoylating isopeptidase 1), a second class od SUMO-related proteases [83]. The gene *ATG9A* (autophagy related 9A) is essential for autophagy, being trafficked to sites of autophagosome formation [84]. Finally, we identified a down-regulation of the *LRRN3* gene in high-fertility boars, with LRRN3 being involved in Ras-MAPK signaling by facilitating internalization of EGF in clathrin-coated vesicles [85]. *TCF21* (transcription factor 21) is a key inactivation factor of the PI3K/AKT signaling pathway and also of matrix metalloproteinases [86]. This is in agreement with previous proteomic results in bull spermatozoa in relation with fertility [87], with *PDZD8* being up-regulated in high-fertility boars. *BAG4* (*BCL2*-associated athanogene 4) was found to be up-regulated in high-fertility boars, forms a complex with annexin 7 and is positively correlated with levels of Hsp70 and Bcl-2, these two last inhibiting the Cytochrome C dependent activation of Caspase-3, and also involved in mitochondrial apoptosis [88]. 

## 4. Materials and Methods 

### 4.1. Ethics Statement 

Animal husbandry and experimental handling were performed in compliance with the European Community (Directive 2010/63/EU) and current Swedish legislation (SJVFS 2017:40). All procedures were performed according to the European Directive 2010/63/EU, 22/09/2010 for animal experiments and current Swedish legislation (SJVFS 2017:40. Date: 06/2016) and approved by the Bioethics Committee of Murcia University (research code: 639/2012) and the “Regional Committee for Ethical Approval of Animal Experiments” (Linköpings Djurförsöksetiska nämnd) in Linköping, Sweden (permits no. 75-12 and no. ID1400. Date: (12/2017)).

The chemicals used in the experiments were of analytical grade. Unless otherwise stated, all reagents were acquired from Sigma-Aldrich (St. Louis, MO, USA).

### 4.2. Ejaculated Spermatozoa

For experiment 1, young mature boars (9–11 mo, *n* = 3) of proven sperm quality (concentration, motility and morphology) of Swedish Landrace breed were recruited from a controlled breeding farm. The animals were individually kept in separate pens at the Translational Medicine Centre (TMC/CBR-3) of Linköping University under controlled temperature and light regimes (12 h:12 h light/dark cycle). For experiment 2, healthy, mature (1–2 years old) boars of Landrace and Large White breeds with proven fertility were housed in a commercial artificial insemination (AI) enterprise (Topigs Norsvin España, Calasparra, Murcia, Spain). All boars were fed with commercial feedstuff well-adjusted to nutritional requirements of adult AI boars, provided with water *ad libitum* and receiving the same management. Throughout all experiments, animals were handled carefully and in such a way as to avoid any unnecessary stress. The sperm-rich fraction (SRF) was collected using the gloved-hand method yielding, in experiment 1 a total of thirty ejaculates (ten ejaculates per boar) and, in experiment 2, 28 ejaculates from 7 different boars (one ejaculate/boar/month). All ejaculates used in the experiments fulfilled the standards of quantity and sperm quality requirements for AI-doses (˃ 200 × 10^6^ spermatozoa/mL, 70% motile spermatozoa, and 75% of morphologically normal sperm). In all cases, spermatozoa were separated from seminal plasma immediately after ejaculate collection by double centrifugation (1500×g at room temperature for 10 min, Rotofix 32A, Hettich Zentrifugen, Tuttlingen, Germany) and the resulting sperm pellets were stored at −80°C (Ultra Low Freezer; Haier Inc., Qingdao, China).

### 4.3. Experimental Design

#### 4.3.1. Experiment 1. Screening of Sperm miRNAs in the Sperm-rich Fraction (SRF) of the Boar Ejaculate

Each collected SRF was evaluated for sperm concentration and motility (velocity and forward progressive motility) using a light microscope (Zeiss, Stockholm Sweden) equipped with a thermal plate (38 °C), positive phase contrast optics (10× objective), a Charge Coupled Device (CCD) camera (UI-1540LE-M-HQ, Ueye, IDS Imaging Development Systems GmbH, Ubersulm, Germany), and the Qualisperm® Software (Biophos SA, Lausanne, Switzerland). Ejaculates with at least 70% motile and 75% morphologically normal-looking spermatozoa immediately after collection were used for the experiments. Ten boar ejaculates (SRF) from each male (*n* = 3) were used for analyze the miRNA expression profile, after RNA isolation.

#### 4.3.2. Experiment 2: Assessment of the Cargo of RNAs in Ejaculated Spermatozoa from Breeding Boars with High- or Low-fertility (as Farrowing Rate and Litter Size) after AI. 

**Figure 4:**
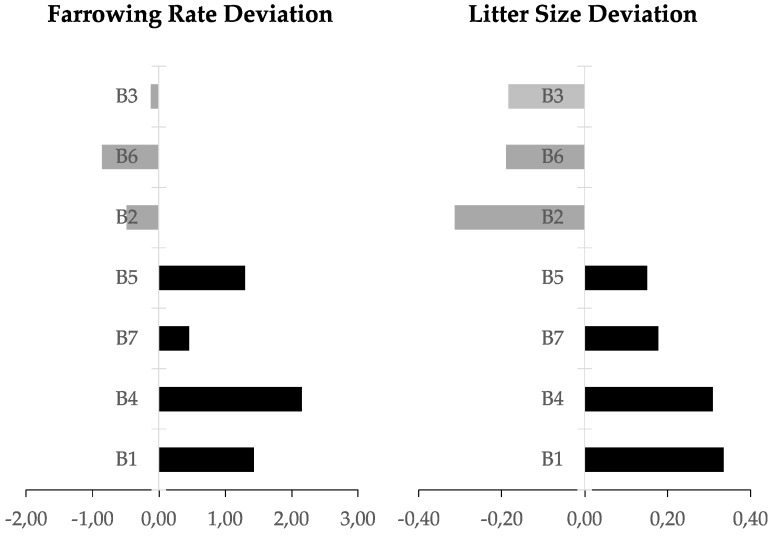
Bar chat of the deviation normalized from the average for each genetic line in terms of boar effect in farrowing rate and litter size.

Ejaculated spermatozoa came from 7 AI boars routinely used in a commercial AI program. Noteworthy, all boars presented normal semen quality, as expected for boars selected for breeding via commercial AI. The ejaculates (*n* = 28) used in the experiment were collected over a period of four months (one ejaculation per boar and month), while in the same period two other ejaculates were collected per week from the same boars and used to inseminate a total of 1722 sows, whose fertility data in terms of farrowing rate and litter size were recorded. The raw fertility data were statistically corrected for parameters related to farm and sow in order to isolate the contribution of the individual boars (direct boar effect [44]). The direct boar effect on fertility was shown as the deviation of both the farrowing rate and litter size of codified selected boars including a correction from the boar population of the same genetic line (Figure 4).

Thus, the 7 boars were classified as having high (*n* = 4) or low (*n* = 3) fertility based in more than 100 artificial inseminations (up to 540) per boar, according to their farrowing rate (FR) and litter size (LS): high-fertility boars with a FR > 0.45 and a LS > 0.15; and low-fertility boars with FR < −0.12 and LS < −0.18. Four ejaculates from each boar were used for analyzing the differential gene expression profile, after RNA isolation.

### 4.4. RNA Isolation

For both experiments, the RNA was obtained using a commercially available kit designed to isolate cell-free RNAs for low quantity samples (miRNeasy kit, Qiagen, Hilden, Germany), following the manufacturer protocol. In brief, each sample (200 μL) was thawed on ice, homogenized and RNA was extracted using 200 μL of QIAzol Lysis Reagent (Qiagen, Hilden, Germany). Samples were mixed by pipetting and incubated (5 min, RT) followed by addition of 200 μL of chloroform. The mixture was shaken vigorously (15 s, RT) and incubated (3 min, RT). Phases were separated by centrifugation (12,000× *g*, 15 min, 4 °C) and the upper aqueous phase (400 μL) was transferred into a gDNA eliminator column (Quiagen), to avoid DNA contamination in the purified RNA. After centrifugation (8000× *g*, 30 s, 4 °C), the resulting phase, free of genomic DNA, were mixed with 600 μL of 100% ethanol by gentle pipetting. A total of 700 μL of sample were transferred into a RNeasy MinElute spin column (Qiagen, Hilden, Germany) in a 2 mL collection tube and centrifuged (8000× *g*, 15 s, RT) in order to allow that RNA bound to the membrane. The flow-through was removed and this step was repeated with the rest of the sample (300 μL) using the same column. Later, the column was sequentially washed with 700 μL of RWT buffer (8000× *g*, 15 s, RT), 500 μL of RPE buffer (8000× *g*, 15 s, RT) and 500 μL of 80% ethanol (8000× *g*, 2 min, RT), discarding the flow-through in each washing. Finally, the column was dried (15,000× *g*, 6 min, RT) and the flow-through was discarded. RNA was eluted by placing 14 μL nuclease-free water in the spin column membrane and centrifuging (15,000× *g*, 1 min, RT). Total RNA content and its quality was determined by NanoDrop^®^ 1000 (Thermo Fisher Scientific, Waltham, MA, USA). Only samples with high RNA quality were snap-frozen in liquid nitrogen and stored at −80 °C for its further analysis. 

### 4.5. MiRNA Microarray Protocol (Experiment 1)

MiRNA expression profiling was carried out using Affymetrix^®^ GeneChip miRNA 4.0 Array (Thermo Fisher Scientific, Göteborg, Sweden), following the manufacturer guidelines. Biotin-labeled RNA was synthesized using Affymetrix^®^ FlashTag^TM^ Biotin HSR RNA Labelling Kit (Thermo Fisher Scientific, Göteborg, Sweden). Briefly, 100 ng of total RNA were subjected to poly-A tail incorporation to 3′-end and, subsequently, a biotin-labeled 3DNA molecule was joined to the 3′-end using DNA ligase. 

### 4.6. Microarray Protocol (Experiment 2)

Total RNA (75 ng) from each sample were used to make cDNA using GeneChip^®^ Whole Transcript Plus reagent kit (Thermo Fisher Scientific, Göteborg, Sweden) following the manufacturer protocol [4].

### 4.7. miRNA Arrays and Microarrays Hybridization and Scanning

Finally, 21.5 μL of biotin-labeled sample (experiment 1: miRNA arrays) or 41 μL of fragmented and labelled single stranded cDNA (experiment 2: microarrays) were mixed with 110.5 μL or 109 μL, respectively, of hybridization master mix to make a cocktail hybridization mix for a single reaction. The hybridization cocktail was then incubated first at 99 °C for 5 min, followed by a descent to 45 °C until loaded on the array chip (GeneChip miRNA 4.0 for miRNA and the Affymetrix GeneChip® Porcine Gene 1.0 ST Array for gene expression; Thermo Fisher Scientific, Gothenburg, Sweden). A total of 130 μL of the cocktail hybridization mix was loaded into each array chip and was incubated at 45 °C under 60 rotations per min, for 18 h (16 h for the microarrays in experiment 2). The hybridized cartridge array chip was then unloaded and subjected to washing and staining using a GeneChip^®^ Fluidics Station 450 (Thermo Fisher Scientific, Göteborg, Sweden), to be finally scanned using the Affymetrix GeneChip^®^ scanner GCS3000 (Thermo Fisher Scientific, Göteborg, Sweden).

### 4.8. Bioinformatics and Statistics

The intensity data of each array chip was processed using the robust multi-array average (RMA) normalization, computing average intensity values by background adjustment, quantile normalization among arrays and finally log_2_ transformation for extracting the expression values of each transcript in the probe set, as implemented in the official Transcriptome Analysis Console (TAC; version 4.0) from Affymetrix. Molecular and biological functions as well as of overrepresentation analysis were identified using PANTHER GO [89].

Statistical analysis was performed by Affymetrix^®^ Transcriptome Analysis Console software (version 4.0, Thermo Fisher Scientific, Göteborg, Sweden). Experiment 1: a pool of 10 ejaculates for each male (*n* = 3) were used for miRNA analysis. The average expression in the arrays (Mean ± Standard Deviation of the log_2_ average expression) were used for describing the differential expression intensities analyzed by the miRNAs array. Experiment 2: one comparation were performed between the high (*n* = 4, and four ejaculates per each boar) and low (*n* = 3, and four ejaculates per each boar) fertility boars. The differential expressed genes (DEGs) that fulfilled the criteria (ANOVA p-value < 0.05) were extracted as significantly up- or down-regulated transcripts. Finally, the analysis of the target genes, with a score of more than 0.9. in the miRDB [80] was used for finding the relation between miRNAs (experiment 1) and transcripts (experiment 2).

## 5. Conclusions

An overrepresentation analysis of the protein class (PANTHER) identified significant fold-increases for C-C chemokine binding (GO:0019957): CCR7 which activates B- and T-lymphocytes, 8-fold increase), XCR1 and CXCR4 (with ubiquitin as natural ligand, 1.24-fold increase), cytokine receptor activity (GO:0005126): IL23R receptor of the IL23 protein, associated to the JAK2 and STAT3, 3.4-fold increase), the TGF-receptor (PC00035) genes ACVR1C and ACVR2B (12-fold increase). Moreover, two micro-RNAs (miR-221 and mir-621) were down- and up-regulated, respectively, in high-fertile males. In conclusion, boars with different fertility performance depict a differential RNA cargo that would be an attractive battery of non-invasive molecular markers predicting fertility.

## Figures and Tables

**Table 1:** Expression (Mean ± Standard Deviation of the log2 average expression or transcript detection) of *Sus scrofa* specific miRNAs detected by the GeneChip™ miRNA 4.0 Array (ThermoFisher Scientific) in spermatozoa retrieved from the sperm-rich fraction (SRF) of the ejaculate of healthy mature boars (*n* = 3). The miRNA is designed to interrogate all mature miRNA sequences in miRBase v20. The array includes 30.424 mature miRNA (all organisms) and we select specifically 10 of the 326 *Sus scrofa*-specific miRNAs included in the array with expression value > 10.

Transcript ID	Expression Mean ± Standard Deviation (log2)	Accession	Sequence Length	Sequence
ssc-miR-1285	13.98 ± 0.13	MIMAT0013954	24	CUGGGCAACAUAGCGAGACCCCGU
ssc-miR-16	12.6 ± 0.74	MIMAT0007754	22	UAGCAGCACGUAAAUAUUGGCG
ssc-miR-4332	12.32 ± 0.29	MIMAT0017962	20	CACGGCCGCCGCCGGGCGCC
ssc-miR-92a	12.06 ± 0.09	MIMAT0013908	22	UAUUGCACUUGUCCCGGCCUGU
ssc-miR-671-5p	11.73 ± 0.54	MIMAT0025381	24	AGGAAGCCCUGGAGGGGCUGGAGG
ssc-miR-4334-5p	11.31 ± 0.05	MIMAT0017966	19	CCCUGGAGUGACGGGGGUG
ssc-miR-425-5p	10.99 ± 0.15	MIMAT0013917	23	AAUGACACGAUCACUCCCGUUGA
ssc-miR-191	10.57 ± 0.22	MIMAT0013876	23	CAACGGAAUCCCAAAAGCAGCUG
ssc-miR-92b-5p	10.53 ± 0.18	MIMAT0017377	24	AGGGACGGGACGCGGUGCAGUGUU
ssc-miR-15b	10.01 ± 0.9	MIMAT0002125	22	UAGCAGCACAUCAUGGUUUACA

**Table 2:** Differential gene expression using a microarrays platform (GeneChip^®^ Porcine Gene 1.0 ST Array) of 77 transcripts (67 transcripts up-regulated and 10 transcripts down-regulated) in high-fertile relative with low-fertile males (Fold change >2 & <–2). Annotation of the full gene name for the transcript and up- (positive fold change) and down-regulation (negative fold change in high-fertile males in comparison to low-fertile males. The Porcine Genome Array provides comprehensive coverage of the *Sus scrofa* transcriptome. The array contains 23,937 probe sets that interrogate approximately 23,256 transcripts from 20,201 *Sus scrofa* genes. The sequence information for this array was selected from public data sources including UniGene GenBank^®^ mRNAs, and GenBank porcine mitochondrial and rRNA sequences.

UP-REGULATED GENES in High-Fertile Boars vs. Low-Fertile Boars	DESCRIPTION	Fold Change	DOWN-REGULATED GENES in High-Fertile Boars vs. Low-Fertile Boars	DESCRIPTION	Fold Change
LOC100739568	replication factor C subunit 1-like	5.72	IFN-DELTA-4; LOC100736862	interferon-delta-4; interferon tau-11-like	−2
AGPAT2	1-acylglycerol-3-phosphate O-acyltransferase 2	3.53	FOXP2; LOC100620234	forkhead box P2; forkhead box protein P2-like	−2.03
FBXW5	F-box and WD repeat domain containing 5	3.52	LOC100155405	olfactory receptor 4K1	−2.03
PPP1R16A	protein phosphatase 1, regulatory subunit 16A	3.34	LOC100516093	olfactory receptor 52N4	−2.27
PIM1	Pim-1 proto-oncogene, serine/threonine kinase	3.26	LOC100517289	E3 ubiquitin-protein ligase RNF220	−2.41
RAB7A	RAB7A, member RAS oncogene family	3.26	LOC100738965; LOC100739445	olfactory receptor 1J4-like	−2.49
C12H17orf97	chromosome 12 open reading frame, human C17orf97	3.1	LOC100525437	ATP synthase subunit d, mitochondrial-like	−3.06
TMEM239; C17H20orf141	transmembrane protein 239; chromosome 17 open reading frame, human C20orf141	3.1	LOC100157815	olfactory receptor 4K15	−3.9
SAE1	SUMO1 activating enzyme subunit 1	3.01	LOC100522664; LOC100622735	olfactory receptor 2AJ1-like	−4.97
TSSK6	testis-specific serine kinase 6	2.91	LOC100157785	olfactory receptor 8H1-like	−5.22
WNT3	wingless-type MMTV integration site family, member 3	2.91			
CARHSP1	calcium regulated heat stable protein 1, 24kDa	2.88			
UBQLNL	ubiquilin-like	2.79			
TSSK2	testis-specific serine kinase 2	2.77			
MAP1LC3A	microtubule-associated protein 1 light chain 3 alpha	2.71			
RABAC1	Rab acceptor 1 (prenylated)	2.68			
PHKG2	phosphorylase kinase, gamma 2 (testis)	2.66			
MTHFSD	methenyltetrahydrofolate synthetase domain containing	2.61			
CSNK1G2	casein kinase 1, gamma 2	2.56			
C9H11orf71	chromosome 9 open reading frame, human C11orf71	2.54			
AGBL5	ATP/GTP binding protein-like 5	2.51			
SGSM2	small G protein signaling modulator 2	2.47			
TIMP2	TIMP metallopeptidase inhibitor 2	2.47			
DUSP18	dual specificity phosphatase 18	2.45			
SARNP	SAP domain containing ribonucleoprotein	2.45			
TNP1	transition protein 1 (during histone to protamine replacement)	2.45			
FAM57A	family with sequence similarity 57, member A	2.44			
EHD1	EH-domain containing 1	2.43			
NSUN4	NOP2/Sun domain family, member 4	2.4			
C7H6orf125	uncharacterized protein C6orf125 homolog	2.39			
EIF4A3; LOC100739660	eukaryotic translation initiation factor 4A3; eukaryotic initiation factor 4A-III-like	2.38			
NDUFA10	NADH dehydrogenase (ubiquinone) 1 alpha subcomplex, 10, 42kDa	2.36			
KHDRBS3; LOC100625959	KH domain containing, RNA binding, signal transduction associated 3; KH domain-containing, RNA-binding, signal transduction-associated protein 3-like	2.33			
CHCHD5	coiled-coil-helix-coiled-coil-helix domain containing 5	2.32			
OSBP2	oxysterol binding protein 2	2.3			
C1H9orf16; LCN2	chromosome 1 open reading frame, human C9orf16; lipocalin 2	2.29			
GRK4	G protein-coupled receptor kinase 4	2.28			
PSMF1; LOC100736938	proteasome inhibitor subunit 1; proteasome inhibitor PI31 subunit	2.28			
CATSPERG	catsper channel auxiliary subunit gamma	2.27			
MFAP3L	microfibrillar-associated protein 3-like	2.26			
TSACC	TSSK6 activating co-chaperone	2.25			
LOC100155534	verprolin-like	2.24			
LOC102166949	E3 ubiquitin-protein ligase RNF169	2.24			
NEURL1	neuralized E3 ubiquitin protein ligase 1	2.24			
FAM46C	family with sequence similarity 46, member C	2.22			
ISG20L2	interferon stimulated exonuclease gene 20kDa-like 2	2.19			
MIEF1	mitochondrial elongation factor 1	2.19			
HTR4; LOC100737835	5-hydroxytryptamine (serotonin) receptor 4, G protein-coupled; 5-hydroxytryptamine receptor 4	2.18			
VRK3	vaccinia related kinase 3	2.16			
CARM1	coactivator-associated arginine methyltransferase 1	2.15			
LOC100152461; LOC100627430; NFYA	nuclear transcription factor Y subunit alpha; nuclear transcription factor Y, alpha	2.15			
LOC100157936; LOC102162592	E3 ubiquitin-protein ligase NRDP1; E3 ubiquitin-protein ligase NRDP1-like	2.13			
LOC102162328	uncharacterized LOC102162328	2.13			
SYCE2	synaptonemal complex central element protein 2	2.12			
UBE2D2	ubiquitin-conjugating enzyme E2D 2	2.12			
LOC100625908; LOC102162228	uncharacterized LOC100625908; uncharacterized LOC102162228	2.1			
ACTR3B	ARP3 actin-related protein 3 homolog B (yeast)	2.09			
CCNY	cyclin Y	2.09			
CPEB3	cytoplasmic polyadenylation element binding protein 3	2.09			
LOC100511504; LOC100515480	maestro heat-like repeat-containing protein family member 1	2.09			
LOC100737397; C1H9orf9	uncharacterized protein C9orf9 homolog; chromosome 1 open reading frame, human C9orf9	2.07			
LOC102158209	uncharacterized LOC102158209	2.04			
REEP2	receptor accessory protein 2	2.04			
TTC7A	tetratricopeptide repeat domain 7A	2.04			
GNA12	guanine nucleotide binding protein (G protein) alpha 12	2.02			
PIM3; BRD1	Pim-3 proto-oncogene, serine/threonine kinase; bromodomain containing 1	2.02			
DESI1	desumoylating isopeptidase 1	2			

**Table 3:** Overrepresentation analysis (PANTHER) of the DEGs between high-fertile and low-fertile boars, according to A. PANTHER molecular function class and B. PANTHER protein class. In both overrepresentation analysis, the expected number of genes in the *Sus scrofa* reference genome is compared with the observed number of genes in the category, resulting in a fold enrichment is indicated in the table (*p* < 0.05 as significant level). The column “Genes IDs” depicts the up-regulated (**bold**) and down-regulated (*italics*) genes between high-fertile boars and low-fertile boars. (*n* = 7).

A. PANTHER Molecular Function Class	Sus Scrofa Ref	Expected	Fold Enrichment	P Value	Gene IDs
C-C chemokine binding (GO:0019957)	24	0.38	7.93	6.76E-03	*CCR7*, *XCR1*, *CXCR4*
chemokine binding (GO:0019956)	24	0.38	7.93	6.76E-03	*CCR7*, *XCR1*, *CXCR4*
cytokine binding (GO:0019955)	88	1.39	4.33	2.98E-03	*CCR7*, *XCR1*, *CXCR4*, *ACVR2B*, *IL23R*
protein binding (GO:0005515)	2824	44.49	1.51	4.32E-04	**WDR34, MTF2, ELOF1, KIF15, KAT7, FGFR2, GPAT3, ATP6V0A2, AGBL5, CCNY,BAG6, TRAF3, EHD1, WDTC1, PPP1R16A, CXCL16, MICU1, AKAP11, MAP1LC3A, SGSM2, DVL1, UBQLNL, ERC1, MOBKL3, SNX14, WNT3, BAG6, NRG4, SHC3, GNA12, UBE2D2, STYX, FIBCD1, TSACC, ATN1**, *CDH10*, *STXBP6*, *TMEFF2*, *CCR7*, *SERPINE2*, *FGD4*, *AKAP7*, *ACVR1C*, *CDSN*, *NUF2*, *ACVR2B*, *PPARGC1B*, *SYT1*, *GDF9*, *ANKRD55*, *GNAT3*, *ANGPTL1*, *WFDC9*, *SYT10*, *IL23R*, *SERPINI2*, *TFPI2*, *CTNNA3*, *XCR1*, *TEAD3, CXCR4*, *BRCC3*, *CDSN*, *CCNJ*
cytokine receptor activity (GO:0005126)	75	1.18	3.39	3.20E-02	*CCR7, XCR1, CXCR4, IL23R*
**B. PANTHER Protein Class**	**Sus Scrofa Ref**	**Expected**	**Fold Enrichment**	**P Value**	**Gene IDs**
TGF-beta receptor (PC00035)	15	0.24	12.69	1.83E-03	*ACVR1C*, *ACVR2B*
serine/threonine protein kinase receptor (PC00205)	20	0.32	9.52	4.10E-03	*ACVR1C, ACVR2B*
protein kinase (PC00193)	266	4.19	2.39	1.05E-02	**TSSK2, TSSK6, RPS6KC1, PRKCQ, VRK3, CSNK1G2, GRK4**, *ACVR1C, ACVR2B*
kinase (PC00137)	386	6.08	1.97	2.10E-02	**TSSK2, TSSK6, NDUFA10, RPS6KC1, PRKCQ, VRK3, CSNK1G2, PI4K2A, GRK4**, *ACVR1C, ACVR2B*
transferase (PC00220)	907	14.29	1.82	2.79E-03	**NTMT1, CHST13, NSUN4, TSSK2, TSSK6, KAT7, GPAT3, METTL2A, GALNT18, NDUFA10, RPS6KC1, PRKCQ, VRK3, FAM177A1, CSNK1G2, CHPT1, PI4K2A, RNGTT, GRK4**, *ACCSL, ME1, ACVR1C, ACVR2B, TGM6*

**Table 4:** Target genes of the 10 miRNA *Sus scrofa*-specific miRNAs detected by the GeneChip™ miRNA 4.0 Array (ThermoFisher Scientific) expressed more than 10 times average expression units (log2 average expression or transcript detection average expression units; experiment 1) in relation to the DEGs using a microarrays platform (GeneChip^®^ Porcine Gene 1.0 ST Array) of 521 transcripts between high- and low-fertile boars (experiment 2). Up-regulated in bold and down-regulated in italics.

miRNA ID	Gene Target	GO – Molecular Function	GO – Biological Process
miR-16	UBQLNL (Ubiquilin-like protein)	GO:0031593 polyubiquitin modification-dependent protein binding	GO:0006511 ubiquitin-dependent protein catabolic process
	CPEB3 (Cytoplasmic polyadenylation element-binding protein 3)	GO:0000900 translation repressor activity, mRNA regulatory element binding	GO:0071230 cellular response to amino acid stimulus GO:0007616 long-term memory
	DESI1 (Desumoylating isopeptidase 1)	GO:0042802 identical protein binding	
	ATG9A (Autophagy-related protein 9A)		GO:0000045 autophagosome assembly
	BAG4 (BAG family molecular chaperone regulator 4)	GO:0031625 ubiquitin protein ligase binding	GO:0071364 cellular response to epidermal growth factor stimulus GO:0045785 positive regulation of cell adhesion
	*LRRN3* *(Leucine-rich repeat neuronal protein 3)*		GO:0051965 positive regulation of synapse assembly
miR-92a	CPEB3 (Cytoplasmic polyadenylation element-binding protein 3)	GO:0000900 translation repressor activity, mRNA regulatory element binding	GO:0071230 cellular response to amino acid stimulus GO:0007616 long-term memory
	TCF21 (ranscription factor 21)	GO:0050681 androgen receptor binding	GO:0060766 negative regulation of androgen receptor signaling pathway
	PDZD8 (PDZ domain-containing protein 8)	GO:0008289 lipid binding	GO:0035556 intracellular signal transduction
miR-15b	UBQLNL (Ubiquilin-like protein)	GO:0031593 polyubiquitin modification-dependent protein binding	GO:0006511 ubiquitin-dependent protein catabolic process
	CPEB3 (Cytoplasmic polyadenylation element-binding protein 3)	GO:0000900 translation repressor activity, mRNA regulatory element binding	GO:0071230 cellular response to amino acid stimulus GO:0007616 long-term memory
	DESI1 (Desumoylating isopeptidase 1)	GO:0042802 identical protein binding	
	ATG9A (Autophagy-related protein 9A)		GO:0000045 autophagosome assembly
	*LRRN3* *(Leucine-rich repeat neuronal protein 3)*		GO:0051965 positive regulation of synapse assembly

## Supplementary Materials

Supplementary materials can be found at https://www.mdpi.com/1422-0067/21/5/1572/s1.
