# Supplementary material for: The Transcriptome of Pig Spermatozoa, and Its Role in Fertility
Source: Int J Mol Sci. 2020 Feb 25;21(5):1572. doi: 10.3390/ijms21051572 (PMC7084236; doi:10.3390/ijms21051572)
Supplement: Supplementary file 1 [file ijms-21-01572-s001.pdf]

Supplementary Table 1. Expression (Mean ± Standard Deviation of the log2 average expression or transcript detection) of *Sus scrofa* specific miRNAs detected by the GeneChip™ miRNA 4.0 Array (ThermoFisher Scientific) in spermatozoa retrieved from the SRF of the ejaculate of healthy mature boars (n=3). The miRNA is designed to interrogate all mature miRNA sequences in miRBase v20. The array includes 30.424 mature miRNA (all organisms) and we select specifically the 326 *Sus scrofa*-specific miRNAs included in the array.

Transcript ID	Expression Mean ± Standard Deviation (log2)	Accession	Sequence Length	Sequence
ssc-miR-1285	13.98 ± 0.13	MIMAT0013954	24	CUGGGCAACAUAGCGAGACCCCGU
ssc-miR-16	12.6 ± 0.74	MIMAT0007754	22	UAGCAGCACGUAAAUAUUGGCG
ssc-miR-4332	12.32 ± 0.29	MIMAT0017962	20	CACGGCCGCCGCCGGGCGCC
ssc-miR-92a	12.06 ± 0.09	MIMAT0013908	22	UAUUGCACUUGUCCCGGCCUGU
ssc-miR-671-5p	11.73 ± 0.54	MIMAT0025381	24	AGGAAGCCCUGGAGGGGCUGGAGG
ssc-miR-4334-5p	11.31 ± 0.05	MIMAT0017966	19	CCCUGGAGUGACGGGGGUG
ssc-miR-425-5p	10.99 ± 0.15	MIMAT0013917	23	AAUGACACGAUCACUCCCGUUGA
ssc-miR-191	10.57 ± 0.22	MIMAT0013876	23	CAACGGAAUCCCAAAAGCAGCUG
ssc-miR-92b-5p	10.53 ± 0.18	MIMAT0017377	24	AGGGACGGGACGCGGUGCAGUGUU
ssc-miR-15b	10.01 ± 0.9	MIMAT0002125	22	UAGCAGCACAUCAUGGUUUACA
ssc-miR-30d	9.89 ± 0.36	MIMAT0013871	24	UGUAAACAUCCCCGACUGGAAGCU
ssc-miR-26a	9.62 ± 0.47	MIMAT0002135	22	UUCAAGUAAUCCAGGAUAGGCU
ssc-miR-484	9.55 ± 0.14	MIMAT0017974	20	CCCAGGGGGCGACCCAGGCU
ssc-miR-103	9.53 ± 0.22	MIMAT0002154	23	AGCAGCAUUGUACAGGGCUAUGA
ssc-miR-296-3p	9.41 ± 0.26	MIMAT0022958	21	AGGGUUGGGCGGAGGCUUUCC
ssc-miR-30c-1-3p	9.37 ± 0.62	MIMAT0022976	20	CUGGGAGAGGGUUGUUUACU
ssc-miR-342	9.28 ± 0.18	MIMAT0013944	25	UCUCACACAGAAAUCGCACCCGUCA
ssc-miR-19b	9.26 ± 0.76	MIMAT0013950	23	UGUGCAAAUCCAUGCAAAACUGA
ssc-miR-17-5p	9.25 ± 0.21	MIMAT0007755	23	CAAAGUGCUUACAGUGCAGGUAG
ssc-miR-28-3p	9.23 ± 0.22	MIMAT0015211	22	CACUAGAUUGUGAGCUCCUGGA
ssc-miR-107	9.2 ± 0.35	MIMAT0002155	23	AGCAGCAUUGUACAGGGCUAUCA
ssc-miR-151-5p	9.17 ± 0.3	MIMAT0013882	21	UCGAGGAGCUCACAGUCUAGU
ssc-miR-125b	9.03 ± 0.77	MIMAT0002120	22	UCCCUGAGACCCUAACUUGUGA
ssc-miR-22-3p	8.99 ± 1.03	MIMAT0015710	22	AAGCUGCCAGUUGAAGAACUGU
ssc-miR-320	8.97 ± 0.38	MIMAT0013878	23	AAAAGCUGGGUUGAGAGGGCGAA
ssc-miR-34c	8.9 ± 0.36	MIMAT0013916	23	AGGCAGUGUAGUUAGCUGAUUGC
ssc-miR-4331	8.85 ± 0.43	MIMAT0017955	22	UGUGGCUGUGGUGUAGGCCAGC
ssc-let-7c	8.83 ± 0.67	MIMAT0002151	22	UGAGGUAGUAGGUUGUAUGGUU
ssc-miR-140-3p	8.7 ± 0.47	MIMAT0006786	23	UACCACAGGGUAGAACCACGGAC
ssc-miR-744	8.56 ± 0.19	MIMAT0015708	22	UGCGGGGCUAGGGCUAACAGCA
ssc-miR-106a	8.44 ± 0.08	MIMAT0002118	24	AAAAGUGCUUACAGUGCAGGUAGC
ssc-miR-132	8.43 ± 0.23	MIMAT0025361	22	UAACAGUCUACAGCCAUGGUCG

ssc-miR-23a	8.39 ± 0.44	MIMAT0002133	21	AUCACAUUGCCAGGGAUUUCC
ssc-miR-489	8.31 ± 0.23	MIMAT0025375	22	AGUGACAUCACAUAUACGGCGG
ssc-miR-30e-5p	8.13 ± 0.88	MIMAT0013872	24	UGUAAACAUCCUUGACUGGAAGCU
ssc-let-7a	8.12 ± 0.71	MIMAT0013865	22	UGAGGUAGUAGGUUGUAUAGUU
ssc-miR-30b-5p	7.89 ± 1.33	MIMAT0007756	22	UGUAAACAUCCUACACUCAGCU
ssc-miR-128	7.81 ± 0.79	MIMAT0002157	21	UCACAGUGAACCGGUCUCUUU
ssc-miR-182	7.75 ± 0.26	MIMAT0025366	24	UUUGGCAAUGGUAGAACUCACACU
ssc-miR-885-3p	7.72 ± 0.19	MIMAT0013903	21	AGGCAGCGGGGUGUAGUGGAU
ssc-miR-20b	7.7 ± 0.36	MIMAT0025359	23	CAAAGUGCUCACAGUGCAGGUAG
ssc-miR-15a	7.68 ± 1.5	MIMAT0007753	21	UAGCAGCACAUAAUGGUUUGU
ssc-miR-30c-5p	7.57 ± 0.67	MIMAT0002167	23	UGUAAACAUCCUACACUCUCAGC
ssc-miR-185	7.54 ± 0.2	MIMAT0007759	22	UGGAGAGAAAGGCAGUUCCUGA
ssc-miR-7137-3p	7.53 ± 0.5	MIMAT0028150	22	AGCUGGUCUGGGAGUUCCCGGG
ssc-miR-151-3p	7.51 ± 0.26	MIMAT0013883	22	CUAGACUGAAGCUCCUUGAGGA
ssc-miR-23b	7.49 ± 0.25	MIMAT0013893	22	AUCACAUUGCCAGGGAUUACCA
ssc-miR-122	7.39 ± 0.62	MIMAT0002119	23	UGGAGUGUGACAAUGGUGUUUGU
ssc-miR-29a	7.39 ± 1.43	MIMAT0013870	23	CUAGCACCAUCUGAAAUCGGUUA
ssc-miR-423-5p	7.38 ± 0.22	MIMAT0013880	23	UGAGGGGCAGAGAGCGAGACUUU
ssc-miR-361-5p	7.36 ± 0.14	MIMAT0013933	22	UUAUCAGAAUCUCCAGGGGUAC
ssc-miR-20a	7.34 ± 0.97	MIMAT0002129	22	UAAAGUGCUUAUAGUGCAGGUA
ssc-let-7d-5p	7.26 ± 0.49	MIMAT0025356	22	AGAGGUAGUAGGUUGCAUAGUU
ssc-miR-130b	7.24 ± 0.39	MIMAT0013922	22	CAGUGCAAUGAUGAAAGGGCAU
ssc-miR-100	7.2 ± 0.65	MIMAT0013911	22	AACCCGUAGAUCCGAACUUGUG
ssc-miR-27a	7.2 ± 0.56	MIMAT0002148	21	UUCACAGUGGCUAAGUUCCGC
ssc-miR-24-3p	7.19 ± 0.43	MIMAT0002134	22	UGGCUCAGUUCAGCAGGAACAG
ssc-miR-193a-5p	7.16 ± 0.16	MIMAT0013894	22	UGGGUCUUUGCGGGCGAGAUGA
ssc-miR-10b	7.04 ± 0.97	MIMAT0013885	22	UACCCUGUAGAACCGAAUUUGU
ssc-miR-99a	6.95 ± 0.63	MIMAT0013896	22	AACCCGUAGAUCCGAUCUUGUG
ssc-miR-92b-3p	6.79 ± 0.06	MIMAT0013909	22	UAUUGCACUCGUCCCGGCCUCC
ssc-miR-874	6.78 ± 0.2	MIMAT0025384	23	CUGCCCUGGCCCGAGGGACCGAC
ssc-miR-27b-3p	6.51 ± 0.8	MIMAT0013890	21	UUCACAGUGGCUAAGUUCUGC
ssc-miR-18a	6.48 ± 0.4	MIMAT0002161	22	UAAGGUGCAUCUAGUGCAGAUA
ssc-miR-146a-5p	6.46 ± 0.95	MIMAT0022963	22	UGAGAACUGAAUUCCAUGGGUU
ssc-miR-30a-5p	6.21 ± 1.54	MIMAT0010193	22	UGUAAACAUCCUCGACUGGAAG
ssc-miR-28-5p	5.87 ± 0.35	MIMAT0002136	22	AAGGAGCUCACAGUCUAUUGAG
ssc-let-7e	5.86 ± 0.3	MIMAT0013866	22	UGAGGUAGGAGGUUGUAUAGUU
ssc-miR-183	5.53 ± 1.46	MIMAT0002145	23	UAUGGCACUGGUAGAAUUCACUG
ssc-miR-181a	5.5 ± 1.07	MIMAT0010191	24	AACAUUCAACGCUGUCGGUGAGUU
ssc-miR-148b-3p	5.48 ± 2.07	MIMAT0013901	22	UCAGUGCAUCACAGAACUUUGU
ssc-miR-7139-3p	5.44 ± 0.32	MIMAT0028154	22	UAGGGCACAGGAUGGGAUGAGG
ssc-miR-210	5.43 ± 0.45	MIMAT0007761	22	CUGUGCGUGUGACAGCGGCUGA
ssc-miR-1307	5.27 ± 0.38	MIMAT0013936	22	ACUCGGCGUGGCGUCGGUCGUG
ssc-miR-4334-3p	5.27 ± 2.05	MIMAT0020366	20	UCCCUGUCCUCCAGGAGCUC
ssc-miR-339	5.2 ± 0.55	MIMAT0025372	21	UCCCUGUCCUCCAGGAGCUCA

ssc-miR-99b	5.19 ± 0.33	MIMAT0006018	22	CACCCGUAGAACCGACCUUGCG
ssc-miR-129b	5.06 ± 0.45	MIMAT0020586	21	CUUUUUGCGGUCUGGGCUUGC
ssc-let-7g	5.02 ± 2.43	MIMAT0013867	22	UGAGGUAGUAGUUUGUACAGUU
ssc-miR-125a	5.02 ± 0.73	MIMAT0013897	23	UCCCUGAGACCCUUUAACCUGUG
ssc-miR-7142-5p	5.01 ± 0.25	MIMAT0028159	23	ACUCUCCGAGGGGCCUUCAAGGG
ssc-miR-365-5p	4.91 ± 0.6	MIMAT0017376	23	GAGGGACUUUCAGGGGCAGCUGU
ssc-miR-425-3p	4.88 ± 0.19	MIMAT0013918	22	AUCGGGAAUGUCGUGUCCGCCC
ssc-miR-1306-3p	4.82 ± 0.04	MIMAT0013938	21	ACGUUGGCUCUGGUGGUGAUG
ssc-miR-339-5p	4.8 ± 0.85	MIMAT0013939	22	UCCCUGUCCUCCAGGAGCUCAC
ssc-miR-221-3p	4.77 ± 0.86	MIMAT0007762	22	AGCUACAUUGUCUGCUGGGUUU
ssc-miR-195	4.75 ± 1.04	MIMAT0013928	21	UAGCAGCACAGAAAUAUUGGC
ssc-miR-370	4.73 ± 0.74	MIMAT0025373	22	GCCUGCUGGGGUGGAACCUGGU
ssc-miR-455-3p	4.56 ± 1.11	MIMAT0013960	21	GCAGUCCAUGGGCAUAUACAC
ssc-miR-7134-3p	4.53 ± 0.21	MIMAT0028144	21	AUGCGGAACCUGCGGAUACGG
ssc-miR-139-5p	4.52 ± 0.73	MIMAT0002159	22	UCUACAGUGCACGUGUCUCCAG
ssc-miR-21	4.48 ± 2.53	MIMAT0002165	22	UAGCUUAUCAGACUGAUGUUGA
ssc-miR-3613	4.47 ± 2.29	MIMAT0025387	20	UGUUGUACUUUUUUUUUUGU
ssc-miR-652	4.39 ± 0.17	MIMAT0017964	26	ACAACCCUAGGAGAGGGUGCCAUUCA
ssc-miR-194a	4.24 ± 1.35	MIMAT0025368	21	UGUAACAGCAACUCCAUGUGG
ssc-let-7i	4.19 ± 2.16	MIMAT0002153	19	UGAGGUAGUAGUUUGUGCU
ssc-miR-574	4.15 ± 0.4	MIMAT0013951	22	CACGCUCAUGCACACACCCACA
ssc-miR-18b	4.08 ± 1.38	MIMAT0020585	23	UAAGGUGCAUCUAGUGCAGUUAG
ssc-miR-664-5p	4.01 ± 0.29	MIMAT0013906	23	CAGGCUAGGAGAAGUGAUUGGAU
ssc-miR-335	3.71 ± 1.92	MIMAT0013955	22	UCAAGAGCAAUAACGAAAAAUG
ssc-miR-222	3.65 ± 1.05	MIMAT0013942	24	AGCUACAUCUGGCUACUGGGUCUC
ssc-miR-331-5p	3.48 ± 0.91	MIMAT0013929	21	UCUAGGUAUGGUCCCAGGGAU
ssc-miR-192	3.4 ± 1.5	MIMAT0013910	21	CUGACCUAUGAAUUGACAGCC
ssc-miR-181d-5p	3.37 ± 0.97	MIMAT0013888	24	AACAUUCAUUGUUGUCGGUGGGUU
ssc-miR-345-3p	3.36 ± 0.63	MIMAT0013900	21	CCCUGAACUAGGGGUCUGGAG
ssc-miR-202-3p	3.34 ± 0.88	MIMAT0022956	19	AGAGGUGUAGGCAUGGGAA
ssc-miR-10a-5p	3.29 ± 1.09	MIMAT0013884	22	UACCCUGUAGAUCCGAAUUUGU
ssc-miR-532-5p	3.25 ± 1.03	MIMAT0013940	22	CAUGCCUUGAGUGUAGGACCGU
ssc-let-7f	3.18 ± 1.88	MIMAT0002152	22	UGAGGUAGUAGAUUGUAUAGUU
ssc-miR-423-3p	3.09 ± 0.93	MIMAT0013881	23	AGCUCGGUCUGAGGCCCCUCAGU
ssc-miR-7140-5p	3.09 ± 0	MIMAT0028155	22	CAACUCAAGGGGGCAUCAUUCA
ssc-miR-499-5p	3.06 ± 1.29	MIMAT0013877	21	UUAAGACUUGCAGUGAUGUUU
ssc-miR-363	3.02 ± 1.18	MIMAT0015711	23	AAUUGCACGGUAUCCAUCUGUAA
ssc-miR-2320-5p	2.87 ± 0.96	MIMAT0020597	22	UGGCACAGGGUCCAGCUGUCGG
ssc-miR-328	2.87 ± 1.34	MIMAT0013949	22	CUGGCCCUCUCUGCCCUUCCGU
ssc-miR-124a	2.73 ± 0.63	MIMAT0002156	21	UAAGGCACGCGGUGAAUGCCA
ssc-miR-7137-5p	2.73 ± 0.35	MIMAT0028149	21	CGGGGGAACUCCCAGACCAGC
ssc-miR-17-3p	2.71 ± 0.86	MIMAT0015268	22	ACUGCAGUGAAGGCACUUGUAG
ssc-miR-486	2.7 ± 0.37	MIMAT0013886	22	UCCUGUACUGAGCUGCCCCGAG
ssc-miR-7138-3p	2.67 ± 0.59	MIMAT0028152	22	GAGGACUGGCCUUGCAGGGUGC

ssc-miR-19a	2.65 ± 0.51	MIMAT0002128	23	UGUGCAAAUCUAUGCAAAACUGA
ssc-miR-148a-3p	2.56 ± 1.13	MIMAT0002124	22	UCAGUGCACUACAGAACUUUGU
ssc-miR-296-5p	2.5 ± 0.22	MIMAT0017952	21	GAGGGCCCCCCCCAAUCCUGU
ssc-miR-429	2.46 ± 1.63	MIMAT0020591	22	UAAUACUGUCUGGUAAUGCCGU
ssc-miR-490	2.42 ± 0.18	MIMAT0025376	22	CAACCUGGAGGACUCCAUGCUG
ssc-miR-490-3p	2.42 ± 0.18	MIMAT0022960	22	CAACCUGGAGGACUCCAUGCUG
ssc-miR-29c	2.39 ± 0.88	MIMAT0002166	22	UAGCACCAUUUGAAAUCGGUUA
ssc-miR-2366	2.32 ± 0.61	MIMAT0025388	22	UGGGUCACAGAAGAGGGUCUGG
ssc-miR-4339	2.31 ± 0.23	MIMAT0017980	24	GCUCUGAGCUGCCCCUCCUCGUCC
ssc-miR-30b-3p	2.3 ± 0.81	MIMAT0015269	21	CUGGGAGGUGGAUGUUUACUU
ssc-miR-421-3p	2.29 ± 0.4	MIMAT0022962	23	AUCAACAGACAUUAAUUGGGCGC
ssc-miR-4335	2.27 ± 0.07	MIMAT0017968	20	GUGCCCAGCGCUGCAGGGCA
ssc-miR-503	2.21 ± 0.33	MIMAT0010189	23	UAGCAGCGGGAACAGUACUGCAG
ssc-miR-671-3p	2.18 ± 0.05	MIMAT0025382	21	UCCGGUUCUCAGGGCUCCACC
ssc-miR-214	2.13 ± 0.31	MIMAT0002147	21	ACAGCAGGCACAGACAGGCAG
ssc-miR-339-3p	2.06 ± 0.54	MIMAT0017381	21	AGCUCCUCGAGGCCAGAGCCC
ssc-miR-885-5p	2.06 ± 0.54	MIMAT0013902	22	UCCAUUACACUACCCUGCCUCU
ssc-miR-1249	2.04 ± 0.4	MIMAT0025385	22	ACGCCCUUCCCCCCCUUCUUCA
ssc-miR-500	2.04 ± 0.97	MIMAT0013956	21	AUGCACCUGGGCAAGGAUUCU
ssc-miR-181d-3p	2.01 ± 0.88	MIMAT0017375	22	CCCACCGAGGGAUGAAUGUCAC
ssc-miR-139-3p	2 ± 0.1	MIMAT0022921	23	UGGAGACGCGGCCCUGUUGGAGU
ssc-miR-205	2 ± 0.83	MIMAT0002146	22	UCCUUCAUUCCACCGGAGUCUG
ssc-miR-378	2 ± 0.4	MIMAT0013868	22	ACUGGACUUGGAGUCAGAAGGC
ssc-miR-184	1.98 ± 0.57	MIMAT0002127	22	UGGACGGAGAACUGAUAAGGGU
ssc-miR-324	1.91 ± 0.46	MIMAT0013957	23	CGCAUCCCCUAGGGCAUUGGUGU
ssc-miR-2320-3p	1.89 ± 0.4	MIMAT0020598	21	CGAUGAUGGUCCCUGUGUUUG
ssc-miR-2483	1.83 ± 0.48	MIMAT0025390	22	AAACAUCUGGUUGGUUGAGAGA
ssc-miR-1306-5p	1.82 ± 0.13	MIMAT0013937	22	CCACCUCCCCUGCAAACGUCCA
ssc-miR-152	1.77 ± 0.69	MIMAT0013887	21	UCAGUGCAUGACAGAACUUGG
ssc-miR-345-5p	1.76 ± 0.02	MIMAT0013899	20	GCUGACUCCUAGUCCAGUGC
ssc-miR-615	1.75 ± 0.55	MIMAT0020595	21	UCCGAGCCUGGGUCUCCCUCU
ssc-miR-181b	1.74 ± 0.76	MIMAT0002126	24	AACAUUCAUUGCUGUCGGUGGGUU
ssc-miR-149	1.71 ± 0.22	MIMAT0018379	23	UCUGGCUCCGUGUCUUCACUCCC
ssc-miR-1343	1.67 ± 0.51	MIMAT0020596	22	CUCCUGGGGCCCGCACUCUCGC
ssc-miR-30c-3p	1.64 ± 0.46	MIMAT0022922	22	CUGGGAGAAGGCUGUUUACUCU
ssc-miR-382	1.58 ± 0.13	MIMAT0017956	21	AAGUUGUUCGUGGUGGAUUCG
ssc-miR-7144-5p	1.58 ± 0.14	MIMAT0028163	22	ACUUUCCCGGGAUUUGGAGCGC
ssc-miR-1839-5p	1.55 ± 0.26	MIMAT0013952	22	AAGGUAGAUAGAACAGGUCUUG
ssc-miR-504	1.55 ± 0.24	MIMAT0013931	23	AGACCCUGGUCUGCACUCUAUCU
ssc-miR-383	1.53 ± 0.28	MIMAT0017972	21	CCACAGCACUGCCUGGUCAGA
ssc-let-7d-3p	1.49 ± 0.65	MIMAT0025357	22	CUAUACGACCUGCUGCCUUUCU
ssc-miR-31	1.48 ± 0.71	MIMAT0025360	22	AGGCAAGAUGCUGGCAUAGCUG
ssc-miR-532-3p	1.46 ± 0.37	MIMAT0013941	22	CCUCCCACACCCAAGGCUUGCA
ssc-miR-1296-5p	1.45 ± 0.16	MIMAT0022964	22	UUAGGGCCCUGGCUCCAUCUCC

ssc-miR-769-3p	1.45 ± 0.43	MIMAT0013958	23	CUGGGAUCUCUGGGGUCUUGGUU
ssc-miR-4338	1.42 ± 0.18	MIMAT0017978	22	AUGUUCAGUCUCAGUGGGAACC
ssc-miR-212	1.41 ± 0.53	MIMAT0025370	23	ACCUUGGCUCUAGACUGCUUACU
ssc-miR-491	1.41 ± 0.24	MIMAT0020592	22	AGUGGGGAACCCUUCCAUGAGG
ssc-miR-22-5p	1.39 ± 0.72	MIMAT0015709	22	AGUUCUUCAGUGGCAAGCUUUA
ssc-miR-490-5p	1.38 ± 0.14	MIMAT0017959	20	CCAUGGAUCCCCAGGUGGGU
ssc-miR-206	1.37 ± 0.32	MIMAT0013864	22	UGGAAUGUAAGGAAGUGUGUGA
ssc-miR-129a	1.33 ± 0.22	MIMAT0013959	22	AAGCCCUUACCCCAAAAAGCAU
ssc-miR-452	1.32 ± 0.14	MIMAT0025374	22	AACUGUUUGCAGAGGAAACUGA
ssc-miR-676-3p	1.32 ± 0.47	MIMAT0013943	21	CCGUCCUAAGGUUGUUGAGUU
ssc-miR-34a	1.3 ± 0.4	MIMAT0007757	22	UGGCAGUGUCUUAGCUGGUUGU
ssc-miR-7136-5p	1.22 ± 0.53	MIMAT0028147	22	UCUGGUCCAGACACUGUGGAGC
ssc-miR-7134-5p	1.21 ± 0.28	MIMAT0028143	21	AUGUCCGCGGGUUCCCUAUCC
ssc-miR-505	1.19 ± 0.16	MIMAT0013961	22	UCAACACUUGCUGGUUUCCUCU
ssc-miR-432-3p	1.17 ± 0.15	MIMAT0017384	20	UGGAUGGCUCCUCCAUGGCU
ssc-miR-7142-3p	1.17 ± 0.36	MIMAT0028160	22	UUUGUUGGCUCCUCUGAAGUGA
ssc-miR-155-5p	1.14 ± 0.06	MIMAT0022959	22	UUAAUGCUAAUUGUGAUAGGGG
ssc-miR-7140-3p	1.14 ± 0.2	MIMAT0028156	22	AUGAUGCCCCUUAGAGUUGAGC
ssc-miR-150	1.12 ± 0.3	MIMAT0025365	22	UCUCCCAACCCUUGUACCAGUG
ssc-miR-130a	1.11 ± 0.44	MIMAT0007758	22	CAGUGCAAUGUUAAAAGGGCAU
ssc-miR-146b	1.09 ± 0.1	MIMAT0010190	21	UGAGAACUGAAUUCCAUAGGC
ssc-miR-30a-3p	1.09 ± 0.33	MIMAT0015300	22	CUUUCAGUCGGAUGUUUGCAGC
ssc-miR-424-3p	1.08 ± 0.14	MIMAT0013921	21	CAAAACGUGAGGCGCUGCUAU
ssc-miR-362	1.06 ± 0.25	MIMAT0017958	25	AAUCCUUGGAACCUAGGUGUGAGUG
ssc-miR-487b	1.04 ± 0.06	MIMAT0017973	22	GUGGUUAUCCCUGUCCUGUUCG
ssc-miR-148a-5p	1.03 ± 0.18	MIMAT0022920	22	AAAGUUCUGAGACACUCCGACU
ssc-miR-497	1.03 ± 0.43	MIMAT0013926	21	CAGCAGCACACUGUGGUUUGU
ssc-miR-301	1.02 ± 0.08	MIMAT0002138	23	CAGUCCAAUAGUAUUGUCAAAGC
ssc-miR-208b	1.01 ± 0.07	MIMAT0013912	22	AUAAGACGAACAAAAGGUUUGU
ssc-miR-331-3p	1.01 ± 0.25	MIMAT0013930	21	GCCCCUGGGCCUAUCCUAGAA
ssc-miR-664-3p	1.01 ± 0.04	MIMAT0013907	24	UAUUCAUUUAUCUCCCAGCCUACA
ssc-miR-126-3p	1 ± 0.3	MIMAT0018378	22	UCGUACCGUGAGUAAUAAUGCG
ssc-miR-1296-3p	1 ± 0.05	MIMAT0017976	22	GAGUGGGGUUUUGACCCUAACC
ssc-miR-7	1 ± 0.59	MIMAT0002141	24	UGGAAGACUAGUGAUUUUGUUGUU
ssc-miR-708-5p	0.99 ± 0.2	MIMAT0013945	23	AAGGAGCUUACAAUCUAGCUGGG
ssc-miR-769-5p	0.99 ± 0.57	MIMAT0020363	21	UGAGACCUCUGGGUUCUGAGC
ssc-miR-30e-3p	0.98 ± 0.1	MIMAT0013873	22	CUUUCAGUCGGAUGUUUACAGC
ssc-miR-361-3p	0.98 ± 0.08	MIMAT0013934	24	CCCCCAGGUGUGAUUCUGAUUUGC
ssc-miR-374a-5p	0.98 ± 0.12	MIMAT0013913	22	UUAUAAUACAACCUGAUAAGUG
ssc-miR-186	0.97 ± 0.05	MIMAT0002162	23	CAAAGAAUUCUCCUUUUGGGCUU
ssc-miR-199b-3p	0.97 ± 0.25	MIMAT0007760	22	UACAGUAGUCUGCACAUUGGUU
ssc-miR-542-5p	0.95 ± 0.23	MIMAT0013924	21	UCGGGGAUCAUCAUGUCACGA
ssc-miR-1271	0.94 ± 0.03	MIMAT0017967	20	UGCCUGCUAUGUGCCAGGCA
ssc-miR-194b-5p	0.94 ± 0.23	MIMAT0020365	22	UGUAACAGCGACUCCAUGUGGA

ssc-miR-432-5p	0.94 ± 0.18	MIMAT0013946	21	UCUUGGAGUAGGUCAUUGGGU
ssc-miR-374a-3p	0.93 ± 0.3	MIMAT0013914	22	CUUAUCAGGUUGUAUUGUAAUU
ssc-miR-27b-5p	0.92 ± 0.12	MIMAT0013889	23	AGAGCUUAGCUGAUUGGUGAACA
ssc-miR-143-5p	0.91 ± 0.06	MIMAT0017374	21	GGUGCAGUGCUGCAUCUCUGG
ssc-miR-24-1-5p	0.91 ± 0	MIMAT0015210	24	GUGCCUACUGAGCUGAAACACAGU
ssc-miR-323	0.91 ± 0.24	MIMAT0002139	22	GCACAUUACACGGUCGACCUCU
ssc-miR-376a-3p	0.91 ± 0.24	MIMAT0015716	21	AUCAUAGAGGAAAAUCCACGU
ssc-miR-551a	0.91 ± 0.08	MIMAT0025379	20	GCGACCCACUCUUGGUUUCC
ssc-miR-7135-3p	0.91 ± 0.16	MIMAT0028146	22	AUCUGUCUGUGUCUCUGAGCAG
ssc-miR-628	0.89 ± 0.32	MIMAT0013953	22	AUGCUGACAUAUUUACUAGAGG
ssc-miR-10a-3p	0.88 ± 0.17	MIMAT0022954	21	CAAAUUCGUAUCUAGGGGAAU
ssc-miR-215	0.88 ± 0.14	MIMAT0010192	21	AUGACCUAUGAAUUGACAGAC
ssc-miR-224	0.87 ± 0.03	MIMAT0002132	23	CAAGUCACUAGUGGUUCCGUUUA
ssc-miR-7141-3p	0.87 ± 0.26	MIMAT0028158	21	UCUUAACGUCCAAACCGUUCC
ssc-miR-148b-5p	0.86 ± 0.62	MIMAT0022955	23	GAAGUUCUGUUAUACACUCAGGC
ssc-miR-181c	0.86 ± 0.34	MIMAT0002144	22	AACAUUCAACCUGUCGGUGAGU
ssc-miR-204	0.86 ± 0.03	MIMAT0002164	22	UUCCCUUUGUCAUCCUAUGCCU
ssc-miR-299	0.86 ± 0.09	MIMAT0015712	21	AUGGUUUACCGUCCCACAUAC
ssc-miR-545-5p	0.86 ± 0.33	MIMAT0020593	21	UCAGUAAAUGUUUAUUGGAUG
ssc-miR-582	0.86 ± 0.08	MIMAT0025380	22	UAACCGGUUGAACAACUGAACC
ssc-miR-7143-3p	0.86 ± 0.12	MIMAT0028162	23	UCUGCACUUGAAGCUGAGACUGA
ssc-miR-7144-3p	0.86 ± 0.06	MIMAT0028164	22	GCUCCUUGUCCCGAGACCGCGA
ssc-miR-1224	0.85 ± 0.33	MIMAT0017965	20	CACCUCCUCUCUCCUCAGGU
ssc-miR-326	0.85 ± 0.03	MIMAT0002140	20	CCUCUGGGCCCUUCCUCCAG
ssc-miR-138	0.84 ± 0.29	MIMAT0025363	21	AGCUGGUGUUGUGAAUCAGGC
ssc-miR-217	0.84 ± 0.17	MIMAT0002131	24	UACUGCAUCAGGAACUGAUUGGAU
ssc-miR-450b-3p	0.84 ± 0.31	MIMAT0017380	21	UUGGGAACAUUUUGCAUCCAU
ssc-miR-424-5p	0.83 ± 0.27	MIMAT0013920	22	CAGCAGCAAUUCAUGUUUUGAA
ssc-miR-676-5p	0.83 ± 0.16	MIMAT0017382	22	CUCUUCAAUCUCAGGACUCGCA
ssc-miR-193a-3p	0.81 ± 0.22	MIMAT0013895	22	AACUGGCCUACAAAGUCCCAGU
ssc-miR-708-3p	0.81 ± 0.13	MIMAT0017383	23	CAACUAGACUGUGAGCUUCUAGA
ssc-miR-101	0.8 ± 0.09	MIMAT0010185	21	UACAGUACUGUGAUAACUGAA
ssc-miR-1468	0.8 ± 0.13	MIMAT0025386	22	CUCCGUUUGCCUGUUUUGCUGA
ssc-miR-196a	0.79 ± 0.03	MIMAT0002163	22	UAGGUAGUUUCAUGUUGUUGGG
ssc-miR-219	0.79 ± 0.5	MIMAT0020590	22	AGAGUUGAGUCUGGACGUCCCG
ssc-miR-142-5p	0.78 ± 0.18	MIMAT0013919	21	CAUAAAGUAGAAAGCACUACU
ssc-miR-451	0.78 ± 0.19	MIMAT0018382	22	AAACCGUUACCAUUACUGAGUU
ssc-miR-7136-3p	0.78 ± 0.04	MIMAT0028148	22	UCUCAGUGUUUGAACCAGAAGC
ssc-miR-153	0.77 ± 0.11	MIMAT0002160	20	UUGCAUAGUCACAAAAGUGA
ssc-miR-199a-3p	0.77 ± 0.13	MIMAT0013875	22	ACAGUAGUCUGCACAUUGGUUA
ssc-miR-133b	0.76 ± 0.16	MIMAT0013869	23	UUUGGUCCCCUUCAACCAGCUAU
ssc-miR-374b-5p	0.76 ± 0.05	MIMAT0013915	22	AUAUAAUACAACCUGCUAAGUG
ssc-miR-381	0.76 ± 0.25	MIMAT0017957	23	AGCGAGGUUGCCCUUUGUAUAUU
ssc-miR-545-3p	0.76 ± 0.07	MIMAT0020594	22	AUCAACAAACAUUUAUUGUGUG

ssc-miR-7143-5p	0.76 ± 0.06	MIMAT0028161	24	AAGCUCAGCUCUGAAGUGCAGAGG
ssc-miR-137	0.75 ± 0.19	MIMAT0025362	23	UUAUUGCUUAAGAAUACGCGUAG
ssc-miR-144	0.75 ± 0.02	MIMAT0025364	19	UACAGUAUAGAUGAUGUAC
ssc-miR-202-5p	0.75 ± 0.14	MIMAT0013948	21	UUCCUAUGCAUAUACUUCUUU
ssc-miR-218	0.75 ± 0.23	MIMAT0025371	21	UUGUGCUUGAUCUAACCAUGU
ssc-miR-218-5p	0.75 ± 0.23	MIMAT0022961	21	UUGUGCUUGAUCUAACCAUGU
ssc-miR-29b	0.75 ± 0.37	MIMAT0002137	23	UAGCACCAUUUGAAAUCAGUGUU
ssc-miR-338	0.75 ± 0.25	MIMAT0015713	22	UCCAGCAUCAGUGAUUUUGUUG
ssc-miR-140-5p	0.74 ± 0.16	MIMAT0002143	21	AGUGGUUUUACCCUAUGGUAG
ssc-miR-450c-5p	0.74 ± 0.12	MIMAT0015717	22	UUUUGCGAUGUGUUCCUAAUAC
ssc-miR-493-5p	0.74 ± 0.05	MIMAT0025377	22	UUGUACAUGGUAGGCUUUCAUU
ssc-miR-9-1	0.74 ± 0.07	MIMAT0002168	23	UCUUUGGUUAUCUAGCUGUAUGA
ssc-miR-9-2	0.74 ± 0.07	MIMAT0002169	23	UCUUUGGUUAUCUAGCUGUAUGA
ssc-miR-135	0.73 ± 0.07	MIMAT0002121	23	UAUGGCUUUUUAUUCCUAUGUGA
ssc-miR-194b-3p	0.73 ± 0.13	MIMAT0017951	22	CCAGUGGAGAUGCUGUUACCUU
ssc-miR-221-5p	0.73 ± 0.13	MIMAT0022949	26	ACCUGGCAUACAAUGUAGAUUUCUGU
ssc-miR-145-5p	0.72 ± 0.05	MIMAT0002123	24	GUCCAGUUUUCCCAGGAAUCCCUU
ssc-miR-7138-5p	0.71 ± 0.04	MIMAT0028151	23	UCCCAGCAAGUGUCCAUCCAUCU
ssc-miR-218b	0.7 ± 0.11	MIMAT0020589	22	UUGUGCUUGAUCUAACCAUGUG
ssc-miR-365-3p	0.7 ± 0.13	MIMAT0013904	22	UAAUGCCCCUAAAAAUCCUUAU
ssc-miR-9	0.7 ± 0.08	MIMAT0025358	22	UCUUUGGUUAUCUAGCUGUAUG
ssc-miR-133a-3p	0.69 ± 0.15	MIMAT0010186	21	UUGGUCCCCUUCAACCAGCUG
ssc-miR-1839-3p	0.69 ± 0.24	MIMAT0017385	21	AGACCUACUUUUCUACCAACA
ssc-miR-187	0.69 ± 0.14	MIMAT0020587	22	UCGUGUCUUGUGUUGCAGCCGG
ssc-miR-196b-3p	0.69 ± 0.37	MIMAT0017379	22	CGACAGCACGACACUGCCUUCA
ssc-miR-325	0.69 ± 0.2	MIMAT0002150	23	CCUAGUAGGUGUUCAGUAAGUGU
ssc-miR-411	0.69 ± 0.1	MIMAT0017954	20	AUGUAACACGGUCCACUAAC
ssc-miR-935	0.69 ± 0.41	MIMAT0013947	23	CCAGUUACCGCUUCCGCUACCGC
ssc-miR-136	0.68 ± 0.09	MIMAT0002158	23	ACUCCAUUUGUUUUGAUGAUGGA
ssc-miR-24-2-5p	0.68 ± 0.07	MIMAT0026468	23	GUGCCUACUGAGCUGAUAUCAGU
ssc-miR-493-3p	0.68 ± 0.06	MIMAT0025378	22	UGAAGGUCUACUGUGUGCCAGG
ssc-miR-494	0.68 ± 0.02	MIMAT0017979	22	AGGUUGUCGUGUUGUCUUCUCU
ssc-miR-758	0.68 ± 0.42	MIMAT0015719	21	UUUGUGACCUGGUCCACUAAC
ssc-miR-143-3p	0.66 ± 0.26	MIMAT0013879	21	UGAGAUGAAGCACUGUAGCUC
ssc-miR-340	0.66 ± 0.14	MIMAT0013891	22	UUAUAAAGCAAUGAGACUGAUU
ssc-miR-450a	0.66 ± 0.18	MIMAT0010188	22	UUUUGCGAUGUGUUCCUAAUAU
ssc-miR-1	0.65 ± 0.04	MIMAT0010187	21	UGGAAUGUAAAGAAGUAUGUA
ssc-miR-105-2	0.65 ± 0.05	MIMAT0002117	20	UCAAAUGCUCAGACUCCUUG
ssc-miR-369	0.65 ± 0.05	MIMAT0015714	21	AAUAAUACAUGGUUGAUCUUU
ssc-miR-450b-5p	0.65 ± 0.16	MIMAT0013927	22	UUUUGCAAUAUGUUCCUGAAUA
ssc-miR-105-1	0.64 ± 0.14	MIMAT0002116	20	UCAAAUGCUCAGACUCCUGU
ssc-miR-2411	0.64 ± 0.38	MIMAT0025389	22	UGGAGUGACUGUCAGAUGCAGC
ssc-miR-455-5p	0.64 ± 0.06	MIMAT0022957	22	UAUGUGCCUUUGGACUACAUCG
ssc-miR-126-5p	0.63 ± 0.12	MIMAT0018377	21	CAUUAUUACUUUUGGUACGCG

ssc-miR-142-3p	0.63 ± 0.12	MIMAT0020362	22	UGUAGUGUUUCCUACUUUAUGG
ssc-miR-146a-3p	0.63 ± 0.03	MIMAT0017971	22	CCUGUGAAGUUUAGUUCUUCAG
ssc-miR-155-3p	0.62 ± 0.14	MIMAT0017953	21	UCCUACAUGUUAGCAUUAACA
ssc-miR-190b	0.62 ± 0.12	MIMAT0020588	22	UGAUAUGUUUGAUAUUGGGUUG
ssc-miR-196b	0.62 ± 0.03	MIMAT0025369	22	UAGGUAGUUUCCUGUUGUUGGG
ssc-miR-196b-5p	0.62 ± 0.03	MIMAT0013923	22	UAGGUAGUUUCCUGUUGUUGGG
ssc-miR-218-3p	0.62 ± 0.06	MIMAT0017969	21	AUGGUUCUGUCAAGCACCAUG
ssc-miR-374b-3p	0.62 ± 0.04	MIMAT0017378	22	CUUAUCAGGUUGUAUUAUCAUU
ssc-miR-376a-5p	0.62 ± 0	MIMAT0015715	23	GUAGAUUCUCCUUCUAUGAGUAC
ssc-miR-450c-3p	0.62 ± 0.06	MIMAT0015718	22	AUUGGGAACAUUUUGCAUUCGU
ssc-miR-499-3p	0.62 ± 0.19	MIMAT0017373	22	AACAUCACAGCAAGUCUGUGCU
ssc-miR-542-3p	0.62 ± 0.11	MIMAT0013925	22	UGUGACAGAUUGAUAACUGAAA
ssc-miR-7135-5p	0.62 ± 0.06	MIMAT0028145	21	UCUCUGAGACACUGACUGUGG
ssc-miR-95	0.62 ± 0.3	MIMAT0002142	22	UUCAACGGGUAUUUAUUGAGCA
ssc-miR-376c	0.61 ± 0.12	MIMAT0017960	22	GUGGAUAUUCCUUCUAUGUUUA
ssc-miR-4337	0.61 ± 0.12	MIMAT0017977	22	AGGGUAUAUAAGCCUUCACUGG
ssc-miR-199b-5p	0.6 ± 0.24	MIMAT0017339	22	CCCAGUGUUUAGACUAUCUGUU
ssc-miR-98	0.6 ± 0.03	MIMAT0013905	22	UGAGGUAGUAAGUUGUAUUGUU
ssc-miR-190a	0.59 ± 0.06	MIMAT0025367	21	UGAUAUGUUUGAUAUAUUAGG
ssc-miR-32	0.59 ± 0.14	MIMAT0002149	21	UAUUGCACAUUACUAAGUUGC
ssc-miR-199a-5p	0.58 ± 0.25	MIMAT0013874	23	CCCAGUGUUCAGACUACCUGUUC
ssc-miR-421-5p	0.58 ± 0.51	MIMAT0017970	24	CCUCAUUAAAUGUUUGUUGAAUGA
ssc-miR-127	0.54 ± 0.13	MIMAT0013932	22	UCGGAUCCGUCUGAGCUUGGCU
ssc-miR-1277	0.54 ± 0.16	MIMAT0013935	22	UACGUAGAUAUAUAUGUAUUUU
ssc-miR-133a-5p	0.54 ± 0.27	MIMAT0015299	22	AGCUGGUAAAAUGGAACCAAAU
ssc-miR-145-3p	0.54 ± 0.19	MIMAT0022919	22	GGAUUCCUGGAAAUACUGUUCU
ssc-miR-376b	0.51 ± 0.04	MIMAT0017961	22	GUGGCUAUUCCUUCUAUGUUUA
ssc-miR-7141-5p	0.51 ± 0.02	MIMAT0028157	21	GACGGUUUGGACGUUAAGAAC
ssc-miR-7139-5p	0.48 ± 0.21	MIMAT0028153	23	CCAUUCCUUCGUCUGUGCACUAG
ssc-miR-216	0.4 ± 0.13	MIMAT0002130	23	UAAUCUCAGCUGGCAACUGUGAG

Supplementary Table 2. Differential gene expression using a microarrays platform (GeneChip® Porcine Gene 1.0 ST Array) of 521 transcripts (347 transcripts up-regulated and 174 transcripts down-regulated) in high-fertile relative with low-fertile males. Annotation of the full gene name for the transcript and up- (positive fold change) and down-regulation (negative fold change in high-fertile males in comparison to low-fertile males. The Porcine Genome Array provides comprehensive coverage of the S. scrofa transcriptome. The array contains 23.937 probe sets that interrogate approximately 23.256 transcripts from 20.201 *Sus scrofa* genes. The sequence information for this array was selected from public data sources including UniGene GenBank® mRNAs. and GenBank porcine mitochondrial and rRNA sequences.

UP-REGULATED GENES in high-fertile boars vs. low-fertile boars	DESCRIPTION	Fold Change	DOWN-REGULATED GENES in high-fertile boars vs. low-fertile boars	DESCRIPTION	Fold Change
LOC100739568	replication factor C subunit 1-like	5.72	LOC102162067	interleukin-6 receptor subunit alpha-like	-1.09
AGPAT2	1-acylglycerol-3-phosphate O-acyltransferase 2	3.53	CRHBP	corticotropin releasing hormone binding protein	-1.1
FBXW5	F-box and WD repeat domain containing 5	3.52	LOC106509016	transmembrane protein 62-like	-1.1
PPP1R16A	protein phosphatase 1. regulatory subunit 16A	3.34	TMEM171	transmembrane protein 171	-1.13
PIM1	Pim-1 proto-oncogene. serine/threonine kinase	3.26	PPP1R26	protein phosphatase 1. regulatory subunit 26	-1.14
RAB7A	RAB7A. member RAS oncogene family	3.26	LOC100155532	olfactory receptor 49-like	-1.17
C12H17orf97	chromosome 12 open reading frame. human C17orf97	3.1	LOC100739080; LOC100517351	mast cell protease 3-like	-1.17
TMEM239; C17H20orf141	transmembrane protein 239; chromosome 17 open reading frame. human C20orf141	3.1	MEF2C; LOC100625305	myocyte enhancer factor 2C; myocyte-specific enhancer factor 2C-like	-1.17
SAE1	SUMO1 activating enzyme subunit 1	3.01	CDSN	corneodesmosin	-1.18
TSSK6	testis-specific serine kinase 6	2.91	GATM	glycine amidinotransferase (L-arginine:glycine amidinotransferase)	-1.18
WNT3	wingless-type MMTV integration site family. member 3	2.91	ME1	malic enzyme 1. NADP(+)-dependent. cytosolic	-1.2
CARHSP1	calcium regulated heat stable protein 1. 24kDa	2.88	RGMB; LOC100525212	repulsive guidance molecule family member b; RGM domain family member B-like	-1.2
UBQLNL	ubiquilin-like	2.79	CBLN3	cerebellin 3 precursor	-1.21
TSSK2	testis-specific serine kinase 2	2.77	GOLM1	golgi membrane protein 1	-1.21
MAP1LC3A	microtubule-associated protein 1 light chain 3 alpha	2.71	INPP4A; LOC102166683	inositol polyphosphate-4-phosphatase type I A; uncharacterized LOC102166683	-1.21
RABAC1	Rab acceptor 1 (prenylated)	2.68	LOC106510685; LOC106510683	desmocollin-2-like; uncharacterized LOC106510683	-1.21
PHKG2	phosphorylase kinase. gamma 2 (testis)	2.66	TWISTNB; LOC106504098	TWIST neighbor; DNA-directed RNA polymerase I subunit RPA43-like	-1.21
MTHFSD	methenyltetrahydrofolate synthetase domain containing	2.61	AKR1C4; AKR1CL1	aldo-keto reductase family 1. member C4 (chlordecone reductase; 3-alpha hydroxysteroid dehydrogenase. type I; dihydrodiol dehydrogenase 4); aldo-keto	-1.22

				reductase family 1. member C-like 1	
CSNK1G2	casein kinase 1. gamma 2	2.56	ANKRD55	ankyrin repeat domain 55	-1.22
C9H11orf71	chromosome 9 open reading frame. human C11orf71	2.54	CATSPERB	catsper channel auxiliary subunit beta	-1.23
AGBL5	ATP/GTP binding protein-like 5	2.51	CMYA5; LOC100738205	cardiomyopathy associated 5; cardiomyopathy-associated protein 5-like	-1.23
SGSM2	small G protein signaling modulator 2	2.47	HR	hair growth associated	-1.23
TIMP2	TIMP metallopeptidase inhibitor 2	2.47	LOC100523158	spatacsin-like	-1.23
DUSP18	dual specificity phosphatase 18	2.45	LOC100737852	peptidyl-prolyl cis-trans isomerase C-like	-1.23
SARNP	SAP domain containing ribonucleoprotein	2.45	CXCR4	chemokine (C-X-C motif) receptor 4	-1.24
TNP1	transition protein 1 (during histone to protamine replacement)	2.45	METTL18	methyltransferase like 18	-1.24
FAM57A	family with sequence similarity 57. member A	2.44	SERPINE2	serpin peptidase inhibitor. clade E (nexin. plasminogen activator inhibitor type 1). member 2	-1.24
EHD1	EH-domain containing 1	2.43	SLC25A4	solute carrier family 25 (mitochondrial carrier; adenine nucleotide translocator). member 4	-1.24
NSUN4	NOP2/Sun domain family. member 4	2.4	STARD9	StAR-related lipid transfer (START) domain containing 9	-1.24
C7H6orf125	uncharacterized protein C6orf125 homolog	2.39	INTS7	integrator complex subunit 7	-1.25
EIF4A3; LOC100739660	eukaryotic translation initiation factor 4A3; eukaryotic initiation factor 4A-III-like	2.38	SPARCL1	SPARC-like 1 (hevin)	-1.25
NDUFA10	NADH dehydrogenase (ubiquinone) 1 alpha subcomplex. 10. 42kDa	2.36	TFPI2	tissue factor pathway inhibitor 2	-1.25
KHDRBS3; LOC100625959	KH domain containing. RNA binding. signal transduction associated 3; KH domain-containing. RNA-binding. signal transduction-associated protein 3-like	2.33	TPRA1	transmembrane protein. adipocyte asscociated 1	-1.25
CHCHD5	coiled-coil-helix-coiled-coil-helix domain containing 5	2.32	ACCS; ACCSL	1-aminocyclopropane-1-carboxylate synthase homolog (Arabidopsis)(non-functional); 1-aminocyclopropane-1-carboxylate synthase homolog (Arabidopsis)(non-functional)-like	-1.26
OSBP2	oxysterol binding protein 2	2.3	C5H12orf60	chromosome 5 open reading frame. human C12orf60	-1.26
C1H9orf16; LCN2	chromosome 1 open reading frame. human C9orf16; lipocalin 2	2.29	FAM161B	family with sequence similarity 161. member B	-1.26
GRK4	G protein-coupled receptor kinase 4	2.28	GJC1	gap junction protein. gamma 1. 45kDa	-1.26
PSMF1; LOC100736938	proteasome inhibitor subunit 1; proteasome inhibitor PI31 subunit	2.28	GJC1	gap junction protein. gamma 1. 45kDa	-1.26
CATSPERG	catsper channel auxiliary subunit gamma	2.27	LOC102164408; BRCC3	uncharacterized LOC102164408; BRCA1/BRCA2-containing complex. subunit 3	-1.26
MFAP3L	microfibrillar-associated protein 3-like	2.26	ZNF283	zinc finger protein 283	-1.26

TSACC	TSSK6 activating co-chaperone	2.25	LOC100515880; LOC100737570	olfactory receptor 2C3-like	-1.27
LOC100155534	verprolin-like	2.24	TMEM38A	transmembrane protein 38A	-1.27
LOC102166949	E3 ubiquitin-protein ligase RNF169	2.24	ACVR1C	activin A receptor. type IC	-1.28
NEURL1	neuralized E3 ubiquitin protein ligase 1	2.24	DDIAS	DNA damage-induced apoptosis suppressor	-1.28
FAM46C	family with sequence similarity 46. member C	2.22	FRMD3	FERM domain containing 3	-1.28
ISG20L2	interferon stimulated exonuclease gene 20kDa-like 2	2.19	LOC100153559; LOC102165373	leucine rich adaptor protein 1-like	-1.28
MIEF1	mitochondrial elongation factor 1	2.19	LOC100522904	nucleoporin NUP53-like	-1.28
HTR4; LOC100737835	5-hydroxytryptamine (serotonin) receptor 4. G protein-coupled; 5-hydroxytryptamine receptor 4	2.18	NUF2	NUF2. NDC80 kinetochore complex component	-1.28
VRK3	vaccinia related kinase 3	2.16	CCNJ	cyclin J	-1.29
CARM1	coactivator-associated arginine methyltransferase 1	2.15	HKDC1	hexokinase domain containing 1	-1.29
LOC100152461; LOC100627430; NFYA	nuclear transcription factor Y subunit alpha; nuclear transcription factor Y. alpha	2.15	TARBP1	TAR (HIV-1) RNA binding protein 1	-1.29
LOC100157936; LOC102162592	E3 ubiquitin-protein ligase NRDP1; E3 ubiquitin-protein ligase NRDP1-like	2.13	MMP19	matrix metallopeptidase 19	-1.3
LOC102162328	uncharacterized LOC102162328	2.13	PIWIL4	piwi-like RNA-mediated gene silencing 4	-1.3
SYCE2	synaptonemal complex central element protein 2	2.12	SLC25A45	solute carrier family 25. member 45	-1.3
UBE2D2	ubiquitin-conjugating enzyme E2D 2	2.12	WDR89	WD repeat domain 89	-1.3
LOC100625908; LOC102162228	uncharacterized LOC100625908; uncharacterized LOC102162228	2.1	XKR7; LOC100525014	XK. Kell blood group complex subunit-related family. member 7; cerebral cavernous malformations 2 protein-like	-1.3
ACTR3B	ARP3 actin-related protein 3 homolog B (yeast)	2.09	ACVR2B	activin A receptor. type IIB	-1.31
CCNY	cyclin Y	2.09	ADAM29	ADAM metallopeptidase domain 29	-1.31
CPEB3	cytoplasmic polyadenylation element binding protein 3	2.09	FGD4	FYVE. RhoGEF and PH domain containing 4	-1.31
LOC100511504; LOC100515480	maestro heat-like repeat-containing protein family member 1	2.09	FGFR4	fibroblast growth factor receptor 4	-1.31
LOC100737397; C1H9orf9	uncharacterized protein C9orf9 homolog; chromosome 1 open reading frame. human C9orf9	2.07	HOGA1	4-hydroxy-2-oxoglutarate aldolase 1	-1.31
LOC102158209	uncharacterized LOC102158209	2.04	LOC100511312	transmembrane emp24 domain-containing protein 9	-1.31
REEP2	receptor accessory protein 2	2.04	LOC100522425	olfactory receptor 5M3-like	-1.31
TTC7A	tetratricopeptide repeat domain 7A	2.04	LOC100623141; APOD	apolipoprotein D-like; apolipoprotein D	-1.31
GNA12	guanine nucleotide binding protein (G protein) alpha 12	2.02	LOC100736865	PLASMODESMATA CALLOSE-BINDING PROTEIN 4-like	-1.31
PIM3; BRD1	Pim-3 proto-oncogene. serine/threonine kinase; bromodomain containing 1	2.02	LOC102162154	uncharacterized LOC102162154	-1.31
DESI1	desumoylating isopeptidase 1	2	MIR221	microRNA mir-221	-1.31
LIPE	lipase. hormone-sensitive	1.99	SNCAIP	synuclein. alpha interacting protein	-1.31

LOC100523970; MAD2L2	mitotic spindle assembly checkpoint protein MAD2B; MAD2 mitotic arrest deficient-like 2 (yeast)	1.99	C7H6orf52	chromosome 7 open reading frame. human C6orf52	-1.32
LPIN1; LOC100620364	lipin 1; phosphatidate phosphatase LPIN1-like	1.98	ORC4	origin recognition complex. subunit 4	-1.32
LOC100513137	phospholipid-transporting ATPase IK	1.97	BET1	Bet1 golgi vesicular membrane trafficking protein	-1.33
LOC102168155; SUGCT; LOC106508407	succinate--hydroxymethylglutarate CoA-transferase; succinyl-CoA:glutarate-CoA transferase; succinate--hydroxymethylglutarate CoA-transferase pseudogene	1.96	C13H3orf18	chromosome 13 open reading frame. human C3orf18	-1.33
LOC100736929	uncharacterized LOC100736929	1.95	GPR183	G protein-coupled receptor 183	-1.33
SPATA3	spermatogenesis associated 3	1.95	PPFIA2; LOC102164936	protein tyrosine phosphatase. receptor type. f polypeptide (PTPRF). interacting protein (liprin). alpha 2; liprin-alpha-2-like	-1.33
ENO3	enolase 3 (beta. muscle)	1.94	ADAM7	ADAM metallopeptidase domain 7	-1.34
LOC100515534	uncharacterized LOC100515534	1.94	LOC100520008	guanine nucleotide-binding protein G(I)/G(S)/G(O) subunit gamma-7-like	-1.34
LOC102163404; KDM5B	lysine-specific demethylase 5B; lysine (K)-specific demethylase 5B	1.93	LOC100739844; LOC100736608; LOC102163327; LOC106507218	semaphorin-3A-like; semaphorin-3A; uncharacterized LOC106507218	-1.34
MOBKL3	MOB family member 4. phocein	1.93	TGM6	transglutaminase 6	-1.34
LOC100154378	cAMP and cAMP-inhibited cGMP 3.5-cyclic phosphodiesterase 10A	1.92	ADIPOR2	adiponectin receptor 2	-1.35
NT5M	5.3-nucleotidase. mitochondrial	1.91	DDO	D-aspartate oxidase	-1.35
ATN1	atrophin 1	1.9	DMBT1	deleted in malignant brain tumors 1	-1.35
CIB1	calcium and integrin binding 1 (calmyrin)	1.9	ETV1	ets variant 1	-1.35
DNAJB6	DnaJ (Hsp40) homolog. subfamily B. member 6	1.88	ITLN2; LOC100736733	intelectin 2; intelectin-2-like	-1.35
KAT7	K(lysine) acetyltransferase 7	1.88	LOC100157427	nesprin-1-like	-1.35
LOC100739821	zinc finger MIZ domain-containing protein 2	1.88	LOC100515441	olfactory receptor 52A5-like	-1.35
L3MBTL2	l(3)mbt-like 2 (Drosophila)	1.87	LOC100522145	taurochenodeoxycholic 6 alpha-hydroxylase-like	-1.35
C7H6orf106	chromosome 7 open reading frame. human C6orf106	1.86	STXBP6	syntaxin binding protein 6 (amisyn)	-1.35
CPA5; LOC100625420	carboxypeptidase A5	1.83	LOC100519107	keratin-associated protein 2-4-like	-1.36
LOC102161765	ral guanine nucleotide dissociation stimulator-like	1.83	NAGS	N-acetylglutamate synthase	-1.36
PRKCQ	protein kinase C. theta	1.83	ST6GALNAC4	ST6 (alpha-N-acetyl-neuraminyl-2.3-beta-galactosyl-1.3)-N-acetylgalactosaminide alpha-2.6-sialyltransferase 4	-1.36
ZNRF4	zinc and ring finger 4	1.83	THNSL1	threonine synthase-like 1 (S. cerevisiae)	-1.36
LOC100156879	cytochrome b-c1 complex subunit 1. mitochondrial	1.82	GRID1; LOC102160253; LOC100739571; LOC100739538	glutamate receptor. ionotropic. delta 1; glutamate receptor ionotropic. delta-1-like	-1.37

KLHL25	kelch-like family member 25	1.81	LOC100525652; LOC100738424	olfactory receptor 51L1-like	-1.37
LOC100620325; ERO1B	ERO1-like protein beta; endoplasmic reticulum oxidoreductase beta	1.81	LOC100526139	discoidin. CUB and LCCL domain-containing protein 2	-1.37
ERI3	ERI1 exoribonuclease family member 3	1.8	LOC106510733	cytochrome P450 4A25-like	-1.37
RFX4	regulatory factor X. 4 (influences HLA class II expression)	1.8	PPAG3	pregnancy-associated glycoprotein 3	-1.37
RPS6KC1	ribosomal protein S6 kinase. 52kDa. polypeptide 1	1.8	MTHFD1L	methylenetetrahydrofolate dehydrogenase (NADP+ dependent) 1-like	-1.38
SMPD2	sphingomyelin phosphodiesterase 2. neutral membrane (neutral sphingomyelinase)	1.8	F2RL2	coagulation factor II (thrombin) receptor-like 2	-1.39
LOC100625374	cyclic nucleotide-binding domain-containing protein 2-like	1.79	LOC106508080; LOC100511983	olfactory receptor 7E24-like; olfactory receptor 18-like	-1.39
DGCR2	DiGeorge syndrome critical region gene 2	1.78	PPARGC1B	peroxisome proliferator-activated receptor gamma. coactivator 1 beta	-1.39
PROCA1; LOC100624269	protein interacting with cyclin A1; protein PROCA1-like	1.78	UBTFL1	upstream binding transcription factor. RNA polymerase I-like 1	-1.39
LOC100521004	SPRY domain-containing SOCS box protein 3-like	1.76	NEUROD1	neuronal differentiation 1	-1.4
STK17A; LOC100621897	serine/threonine kinase 17a; serine/threonine-protein kinase 17A	1.75	PRRG4	proline rich Gla (G-carboxyglutamic acid) 4 (transmembrane)	-1.4
ATG9A	autophagy related 9A	1.74	CHRM5	cholinergic receptor. muscarinic 5	-1.41
DCDC2C	doublecortin domain containing 2C	1.73	LOC100516987	olfactory receptor 52H1	-1.41
LOC100512674	zinc finger protein 367	1.73	LOC100739412	histidine decarboxylase-like	-1.41
BANF2	barrier to autointegration factor 2	1.72	SERPINI2	serpin peptidase inhibitor. clade I (pancpin). member 2	-1.41
HMGXB3	HMG box domain containing 3	1.72	SLITRK5	SLIT and NTRK-like family. member 5	-1.41
UBE2J2	ubiquitin-conjugating enzyme E2. J2	1.72	DEFB123	defensin. beta 123	-1.43
CHST13	carbohydrate (chondroitin 4) sulfotransferase 13	1.7	HFM1	HFM1. ATP-dependent DNA helicase homolog	-1.43
LIN52	lin-52 DREAM MuvB core complex component	1.7	LOC100620198	T-cell receptor-associated transmembrane adapter 1	-1.43
LOC100152291	uncharacterized LOC100152291	1.7	ITGB8	integrin. beta 8	-1.44
POC1A	POC1 centriolar protein A	1.7	OLF42-3	olfactory receptor-like protein-like	-1.44
UBXN6	UBX domain protein 6	1.7	TMEFF2	transmembrane protein with EGF-like and two follistatin-like domains 2	-1.44
CTXN1	cortexin 1	1.69	CFTR	cystic fibrosis transmembrane conductance regulator (ATP-binding cassette sub-family C. member 7)	-1.45
SLC25A33; LOC106510614	solute carrier family 25 (pyrimidine nucleotide carrier). member 33; uncharacterized LOC106510614	1.69	IFNK	interferon. kappa	-1.45
CBFA2T3	core-binding factor. runt domain. alpha subunit 2; translocated to. 3	1.68	XCR1	chemokine (C motif) receptor 1	-1.45
CECR2	cat eye syndrome chromosome region. candidate 2	1.67	CXHXorf66	chromosome X open reading frame. human CXorf66	-1.46
LOC100522627	olfactory receptor 10R2-like	1.67	FEZF2; LOC100622205	FEZ family zinc finger 2; fez family zinc finger protein 2	-1.46

LMBR1; LOC102165610	limb development membrane protein 1; uncharacterized LOC102165610	1.65	GDA	guanine deaminase	-1.46
LOC100511053; LOC100624021	zinc finger protein PLAGL2	1.65	KLRF1	killer cell lectin-like receptor subfamily F. member 1	-1.46
FAM166A	family with sequence similarity 166. member A	1.63	GNAT3	guanine nucleotide binding protein. alpha transducing 3	-1.47
JOSD2	Josephin domain containing 2	1.63	ANGPTL1	angiopoietin-like 1	-1.48
LOC100153504; LOC100625760	coagulation factor XIII A chain; coagulation factor XIII A chain-like	1.63	GLI1; LOC100621250	GLI family zinc finger 1; zinc finger protein GLI1-like	-1.48
PPHLN1	periphilin 1	1.63	RFPL4B	ret finger protein-like 4B	-1.48
TTC7B; LOC100738752; LOC102162885	tetratricopeptide repeat domain 7B; tetratricopeptide repeat protein 7B-like; uncharacterized LOC102162885	1.63	TEX26	testis expressed 26	-1.49
FGFR2	fibroblast growth factor receptor 2	1.62	GDF9	growth differentiation factor 9	-1.5
FMR1NB	fragile X mental retardation 1 neighbor	1.62	ARL15	ADP-ribosylation factor-like 15	-1.51
LOC100154280	sex comb on midleg-like protein 4	1.62	CTNNA3	catenin (cadherin-associated protein). alpha 3	-1.52
LOC100517569; LOC100154999	lysine-specific demethylase 4C-like; lysine-specific demethylase 4C	1.62	DLG5	discs. large homolog 5 (Drosophila)	-1.52
POLB	polymerase (DNA directed). beta	1.62	LOC100623462	olfactory receptor 1J4-like	-1.53
PRR30	proline rich 30	1.62	LOC100736882	interferon lambda receptor 1-like	-1.53
GMDS	GDP-mannose 4.6-dehydratase	1.61	SYT1	synaptotagmin I	-1.55
POU3F2	POU class 3 homeobox 2	1.61	FBXO30	F-box protein 30	-1.56
SEC61A2; LOC100627954	Sec61 translocon alpha 2 subunit; protein transport protein Sec61 subunit alpha isoform 2-like	1.61	TNFSF10	tumor necrosis factor (ligand) superfamily. member 10	-1.56
ZFAND3	zinc finger. AN1-type domain 3	1.61	IFIT3	interferon-induced protein with tetratricopeptide repeats 3	-1.58
ELOF1	ELF1 homolog. elongation factor 1	1.6	LOC100517243	adipogenesis regulatory factor	-1.58
FKBP8	FK506 binding protein 8. 38kDa	1.6	USH2A	Usher syndrome 2A (autosomal recessive. mild)	-1.58
GPAT3	glycerol-3-phosphate acyltransferase 3	1.6	LOC100151788	dimethylaniline monooxygenase [N-oxide-forming] 5-like	-1.59
KLC1	kinesin light chain 1	1.6	LPAR6	lysophosphatidic acid receptor 6	-1.59
LOC100522038	poly(ADP-ribose) glycohydrolase	1.6	LOC100156546	olfactory receptor 12D3	-1.6
RNF32	ring finger protein 32	1.6	FAM19A3	family with sequence similarity 19 (chemokine (C-C motif)-like). member A3	-1.62
KCTD9	potassium channel tetramerization domain containing 9	1.59	IL23R	interleukin 23 receptor	-1.62
SH2D4A	SH2 domain containing 4A	1.59	LOC100523309	olfactory receptor 6K3-like	-1.62
ERC1	ELKS/RAB6-interacting/CAST family member 1	1.58	CDH10	cadherin 10. type 2 (T2-cadherin)	-1.63
USP2	ubiquitin specific peptidase 2	1.58	TMEM39A	transmembrane protein 39A	-1.63
ZNF25	zinc finger protein 25	1.58	UBD	ubiquitin D	-1.63
DOLPP1	dolichyldiphosphatase 1	1.57	LOC100157958	laminin subunit gamma-3	-1.66
NRG4	neuregulin 4	1.57	AKAP7	A kinase (PRKA) anchor protein 7	-1.69
AP1M1	adaptor-related protein complex 1. mu 1 subunit	1.56	GYPA	glycophorin A (MNS blood group)	-1.69
ASGR2	asialoglycoprotein receptor 2	1.56	TEAD3	TEA domain family member 3	-1.69
CEP128	centrosomal protein 128kDa	1.56	LOC100519039; LOC100737158	olfactory receptor 51V1-like	-1.7
FAM63A	family with sequence similarity 63. member A	1.56	LOC100157508	olfactory receptor 4K13	-1.72

ENTPD5	ectonucleoside triphosphate diphosphohydrolase 5	1.55	RAB38	RAB38. member RAS oncogene family	-1.73
CLDN4	claudin 4	1.54	SYT10	synaptotagmin X	-1.75
TWSG1	twisted gastrulation BMP signaling modulator 1	1.54	LOC100516985	olfactory receptor 4L1-like	-1.78
C2H11orf94	chromosome 2 open reading frame. human C11orf94	1.53	WFDC9	WAP four-disulfide core domain 9	-1.78
LOC100524756	cell adhesion molecule 1	1.53	LOC100157798	olfactory receptor 4N4	-1.79
ARL9	ADP-ribosylation factor-like 9	1.52	LRRN3	leucine rich repeat neuronal 3	-1.79
LOC100516692; LOC100737702; LOC100516509	testis-expressed sequence 29 protein; rho guanine nucleotide exchange factor 7; rho guanine nucleotide exchange factor 7-like	1.52	LOC106504870	caspase-12-like	-1.82
CXCL16	chemokine (C-X-C motif) ligand 16	1.51	CCDC102B	coiled-coil domain containing 102B	-1.84
LOC100625074; LOC102162253	uncharacterized protein KIAA0895-like	1.51	LOC100522603	olfactory receptor 8K3-like	-1.89
LOC100625124; LOC100154036	MICAL-like protein 1	1.51	CCR7	chemokine (C-C motif) receptor 7	-1.9
NSUN6	NOP2/Sun domain family. member 6	1.51	LOC100523626	olfactory receptor 56B1	-1.94
PAQR7; LOC100737469	progestin and adipoQ receptor family member VII; membrane progestin receptor alpha	1.51	IFN-DELTA-4; LOC100736862	interferon-delta-4; interferon tau-11-like	-2
SLC25A46	solute carrier family 25. member 46	1.51	FOXP2; LOC100620234	forkhead box P2; forkhead box protein P2-like	-2.03
ANKRD35	ankyrin repeat domain 35	1.5	LOC100155405	olfactory receptor 4K1	-2.03
LOC100622412	histone H3	1.5	LOC100516093	olfactory receptor 52N4	-2.27
LOC102160066; LOC102160158	uncharacterized LOC102160066; uncharacterized LOC102160158	1.5	LOC100517289	E3 ubiquitin-protein ligase RNF220	-2.41
TRAPPC9	trafficking protein particle complex 9	1.5	LOC100738965; LOC100739445	olfactory receptor 1J4-like	-2.49
EXD1; LOC100621282	exonuclease 3-5 domain containing 1; exonuclease 3-5 domain-containing protein 1-like	1.49	LOC100525437	ATP synthase subunit d. mitochondrial-like	-3.06
NANOS1	nanos homolog 1 (Drosophila)	1.49	LOC100157815	olfactory receptor 4K15	-3.9
SYNGR4	synaptogyrin 4	1.49	LOC100522664; LOC100622735	olfactory receptor 2AJ1-like	-4.97
USP12	ubiquitin specific peptidase 12	1.49	LOC100157785	olfactory receptor 8H1-like	-5.22
ANKRD42	ankyrin repeat domain 42	1.48			
C4H1orf194	chromosome 4 open reading frame. human C1orf194	1.48			
LOC100155148	mitochondrial intermediate peptidase	1.48			
PRMT8	protein arginine methyltransferase 8	1.48			
WBSCR22	Williams Beuren syndrome chromosome region 22	1.48			
WDTC1	WD and tetratricopeptide repeats 1	1.48			
ZPBP	zona pellucida binding protein	1.48			
C7H6orf136	chromosome 7 open reading frame. human C6orf136	1.47			
FAM160A2	family with sequence similarity 160. member A2	1.47			
LOC100516656; LOC100516841	3-hydroxyisobutyrate dehydrogenase. mitochondrial	1.47			
MEMO1	mediator of cell motility 1	1.47			
PUS7; LOC100627719	pseudouridylate synthase 7 (putative); pseudouridylate synthase 7 homolog	1.47			
FAM214B	family with sequence similarity 214. member B	1.46			

LOC100626951	olfactory receptor 51V1-like	1.46			
LOC102164918; SHC3	uncharacterized LOC102164918; SHC (Src homology 2 domain containing) transforming protein 3	1.46			
LOC102166192	TOX high mobility group box family member 2	1.46			
LOC106504100; LOC100525714	protein phosphatase 1D-like; protein phosphatase 1D	1.46			
PI4K2A	phosphatidylinositol 4-kinase type 2 alpha	1.46			
B4GALT7	xylosylprotein beta 1.4-galactosyltransferase. polypeptide 7	1.45			
BAG6	BCL2-associated athanogene 6	1.45			
LOC100512328; LOC102165593; LOC102166001; LOC102165718	DENN domain-containing protein 1A; DENN domain-containing protein 1A-like; uncharacterized LOC102165718	1.45			
MLLT11	myeloid/lymphoid or mixed-lineage leukemia; translocated to. 11	1.45			
PARN	poly(A)-specific ribonuclease	1.45			
REPS2; LOC100153056	RALBP1 associated Eps domain containing 2; ralBP1-associated Eps domain-containing protein 2-like	1.45			
SETX	senataxin	1.45			
LOC100739624; LOC100739655; LOC102164684; LOC106508498	syntaxin-binding protein 4; syntaxin-binding protein 4-like	1.44			
MLH3	mutL homolog 3	1.44			
UGT1A1	UDP glucuronosyltransferase 1 family. polypeptide A1	1.44			
CTDNEP1	CTD nuclear envelope phosphatase 1	1.43			
DAP3; LOC100625274	death associated protein 3; 28S ribosomal protein S29. mitochondrial-like	1.43			
FKBP6	FK506 binding protein 6. 36kDa	1.43			
ART3	ADP-ribosyltransferase 3	1.42			
C16H5orf49	chromosome 16 open reading frame. human C5orf49	1.42			
GALNT18	polypeptide N-acetylgalactosaminyltransferase 18	1.42			
LIMS2	LIM and senescent cell antigen-like domains 2	1.42			
RUNDC3A	RUN domain containing 3A	1.42			
WDR34	WD repeat domain 34	1.42			
FGF9	fibroblast growth factor 9	1.41			
FGFR1OP	FGFR1 oncogene partner	1.41			
KLHL3	kelch-like family member 3	1.41			
LRRC51	leucine-rich repeat-containing protein 51	1.41			
GANC	glucosidase. alpha; neutral C	1.4			
LMNB1	lamin B1	1.4			
PSMB8	proteasome subunit beta 8; proteasome (prosome. macropain) subunit. beta	1.4			

	type. 8 (large multifunctional peptidase 7)				
ZDHHC7	zinc finger. DHHC-type containing 7	1.4			
BOLA1	bolA family member 1	1.39			
CPA4	carboxypeptidase A4	1.39			
ILKAP	integrin-linked kinase-associated serine/threonine phosphatase	1.39			
NTMT1	N-terminal Xaa-Pro-Lys N-methyltransferase 1	1.39			
PCYT1A	phosphate cytidylyltransferase 1. choline. alpha	1.39			
SOWAHD	sosondowah ankyrin repeat domain family member D	1.39			
TMEM8A	transmembrane protein 8A	1.39			
TRAF3	TNF receptor-associated factor 3	1.39			
FLAD1	flavin adenine dinucleotide synthetase 1	1.38			
LOC100154202	mitogen-activated protein kinase kinase kinase 4	1.38			
NSDHL	NAD(P) dependent steroid dehydrogenase-like	1.38			
SCML2	sex comb on midleg-like 2 (Drosophila)	1.38			
TECR	trans-2.3-enoyl-CoA reductase	1.38			
ZNF268	zinc finger protein 268	1.38			
TCF21	transcription factor 21	1.37			
ZNHIT6	zinc finger. HIT-type containing 6	1.37			
ARL2BP	ADP-ribosylation factor-like 2 binding protein	1.36			
LOC100524488	nucleotide exchange factor SIL1-like	1.36			
LOC102161486	ubiquitin carboxyl-terminal hydrolase 32-like	1.36			
NOD1	nucleotide-binding oligomerization domain containing 1	1.35			
PDIA3	protein disulfide isomerase family A. member 3	1.35			
TMEM27	transmembrane protein 27	1.35			
TRAPPC2L	trafficking protein particle complex 2-like	1.35			
WDR27; LOC102162401; LOC102161809	WD repeat domain 27; uncharacterized LOC102162401; uncharacterized LOC102161809	1.35			
AP2A1	adaptor-related protein complex 2. alpha 1 subunit	1.34			
FUT8	fucosyltransferase 8 (alpha (1.6) fucosyltransferase)	1.34			
KIF15	kinesin family member 15	1.34			
LOC100737129; LOC100512723	GTP-binding protein 2-like	1.34			
LOC106508607	uncharacterized LOC106508607	1.34			
LOC106509520; LOC100517752; LOC100515712	colorectal mutant cancer protein-like; colorectal mutant cancer protein	1.34			
MGAT4B	mannosyl (alpha-1.3-)-glycoprotein beta-1.4-N-	1.34			

	acetylglucosaminyltransferase. isozyme B				
RAD51C	RAD51 paralog C	1.34			
BARX2	BARX homeobox 2	1.33			
BTBD9	BTB (POZ) domain containing 9	1.33			
DNAAF1; LOC100737963	dynein. axonemal. assembly factor 1; uncharacterized LOC100737963	1.33			
FAM177A1	family with sequence similarity 177. member A1	1.33			
LOC100524199; LOC100620736	transcription factor 25	1.33			
P14ARF	p14ARF protein	1.33			
ZNF568	zinc finger protein 568	1.33			
COPS7A	COP9 signalosome subunit 7A	1.32			
FGFR2	fibroblast growth factor receptor 2	1.32			
LCORL	ligand dependent nuclear receptor corepressor-like	1.32			
LOC100157862	sortilin	1.32			
LOC102166011; RNGTT	uncharacterized LOC102166011; RNA guanylyltransferase and 5-phosphatase	1.32			
MORN5	MORN repeat containing 5	1.32			
NMNAT1; LOC100622997	nicotinamide nucleotide adenylyltransferase 1; nicotinamide/nicotinic acid mononucleotide adenylyltransferase 1-like	1.32			
PDZD8	PDZ domain containing 8	1.32			
RNF125	ring finger protein 125. E3 ubiquitin protein ligase	1.32			
WFDC3	WAP four-disulfide core domain 3	1.32			
C17H20orf27	chromosome 17 open reading frame. human C20orf27	1.31			
SH3GL1	SH3-domain GRB2-like 1	1.31			
CNGA4	cyclic nucleotide gated channel alpha 4	1.3			
GMCL1; LOC100625379	germ cell-less. spermatogenesis associated 1; germ cell-less protein-like 1	1.3			
LOC102163983	zinc finger protein 341	1.3			
NUP214	nucleoporin 214kDa	1.3			
LOC100512967; LOC106510605	tumor protein p73-like	1.29			
LOC100516196; LOC106507553	TATA box-binding protein-associated factor RNA polymerase I subunit C; uncharacterized LOC106507553	1.29			
RTN3	reticulon 3	1.29			
TRMT1; LOC100524134; LOC100623538	tRNA methyltransferase 1 homolog (S. cerevisiae); TRM1 tRNA methyltransferase 1 homolog (S. cerevisiae)-like; tRNA (guanine(26)-N(2))-dimethyltransferase-like	1.29			
ATP6V0A2	ATPase. H+ transporting. lysosomal V0 subunit a2	1.28			
ELAVL2	ELAV like neuron-specific RNA binding protein 2	1.28			
FUS	FUS RNA binding protein	1.28			

KHDC3L; LOC100153536	KH domain containing 3-like. subcortical maternal complex member; KHDC3-like protein	1.28			
MDM4	MDM4. p53 regulator	1.28			
SNX14	sorting nexin 14	1.28			
AKAP11	A kinase (PRKA) anchor protein 11	1.27			
CSNK1G1; LOC102166996	casein kinase 1. gamma 1; uncharacterized LOC102166996	1.27			
FAM92A1	family with sequence similarity 92. member A1	1.27			
FIBCD1	fibrinogen C domain containing 1	1.27			
METTL2A	methyltransferase like 2A	1.27			
MIR615	microRNA mir-615	1.27			
NARFL	nuclear prelamin A recognition factor-like	1.27			
VMAC	vimentin-type intermediate filament associated coiled-coil protein	1.27			
BCKDHA	branched chain keto acid dehydrogenase E1. alpha polypeptide	1.26			
CENPA	centromere protein A	1.26			
DUSP1	dual specificity phosphatase 1	1.26			
KCNA3	potassium channel. voltage gated shaker related subfamily A. member 3	1.26			
KCNIP3	Kv channel interacting protein 3. calsenilin	1.26			
KLHDC3	kelch domain containing 3	1.26			
KRI1	KRI1 homolog	1.26			
NLGN3; LOC100156236	neuroligin 3; neuroligin-3	1.26			
TRIM72	tripartite motif containing 72. E3 ubiquitin protein ligase	1.26			
UBR7	ubiquitin protein ligase E3 component n-recognin 7 (putative)	1.26			
APOO	apolipoprotein O	1.25			
ATP4B	ATPase. H+/K+ exchanging. beta polypeptide	1.25			
CRYBB3	crystallin. beta B3	1.25			
FGF14	fibroblast growth factor 14	1.25			
LOC106505929	lysine-specific demethylase 2B-like	1.25			
PACRGL; LOC100623618	PARK2 co-regulated-like; PACRG-like protein	1.25			
IRF3	interferon regulatory factor 3	1.24			
LAPTM4B	lysosomal protein transmembrane 4 beta	1.24			
LOC100738644	leishmanolysin-like peptidase	1.24			
PRMT8	protein arginine methyltransferase 8	1.24			
ANKS3	ankyrin repeat and sterile alpha motif domain containing 3	1.23			
C7H6orf136	chromosome 7 open reading frame. human C6orf136	1.23			
LACE1	lactation elevated 1	1.23			
LOC100738704; LOC106504099	TNFAIP3-interacting protein 3; TNFAIP3-interacting protein 3-like	1.23			
LOC102160001	serine protease inhibitor Kazal-type 6	1.23			

STYX	serine/threonine/tyrosine interacting protein	1.23			
MBTPS2	membrane-bound transcription factor peptidase. site 2	1.22			
PPP3CC; LOC100623062	protein phosphatase 3. catalytic subunit. gamma isozyme; serine/threonine-protein phosphatase 2B catalytic subunit gamma isoform-like	1.22			
SLC2A11	solute carrier family 2 (facilitated glucose transporter). member 11	1.22			
ARHGAP39; LOC100155247	Rho GTPase activating protein 39; rho GTPase-activating protein 39	1.21			
C9H11orf42	chromosome 9 open reading frame. human C11orf42	1.21			
MAP4K2	mitogen-activated protein kinase kinase kinase kinase 2	1.21			
MTSS1L	metastasis suppressor 1-like	1.21			
TRAM2	translocation associated membrane protein 2	1.21			
ATXN7L3	ataxin 7-like 3	1.2			
BAG4	BCL2-associated athanogene 4	1.2			
CHPT1	choline phosphotransferase 1	1.2			
PPARA; LOC106508632	peroxisome proliferator-activated receptor alpha; peroxisome proliferator-activated receptor alpha-like	1.2			
LOC102161647	trichohyalin	1.19			
MTF2	metal response element binding transcription factor 2	1.19			
ACTL7B	actin-like 7B	1.18			
C3H16orf58; LOC106508701	chromosome 3 open reading frame. human C16orf58; uncharacterized LOC106508701	1.18			
LOC102165039	Ig-like V-type domain-containing protein FAM187A	1.18			
LOC100157935	cytochrome c oxidase subunit 8C. mitochondrial	1.17			
MXD3	MAX dimerization protein 3	1.17			
ADSL	adenylosuccinate lyase	1.16			
DVL1	dishevelled segment polarity protein 1	1.16			
COLGALT1	collagen beta(1-O)galactosyltransferase 1	1.14			
KCNH4; LOC100620256	potassium channel. voltage gated eag related subfamily H. member 4; potassium voltage-gated channel subfamily H member 4-like	1.14			
LOC102159765	voltage-dependent P/Q-type calcium channel subunit alpha-1A-like	1.14			
LOC733635	aldo-keto reductase family 1 member C2-like	1.14			
MICU1	mitochondrial calcium uptake 1	1.14			
GNPAT	glyceronephosphate O-acyltransferase	1.12			

FAM109A	family with sequence similarity 109. member A	1.11			
DLGAP2	discs. large (Drosophila) homolog-associated protein 2	1.1			
